# Cervical cerclage versus cervical pessary with or without vaginal progesterone for preterm birth prevention in twin pregnancies and a short cervix: A two-by-two factorial randomised clinical trial

**DOI:** 10.1371/journal.pmed.1004526

**Published:** 2025-02-21

**Authors:** Yen T. N. He, Ha N. H. Pham, Tri C. Nguyen, Trung Q. Bui, Nhu T. Vuong, Diem T. N. Nguyen, Thanh V. Le, Wentao Li, Cam H. Le, Tuong M. Ho, Ben W. Mol, Vinh Q. Dang, Lan N. Vuong

**Affiliations:** 1 Department of Obstetrics and Gynecology, My Duc Phu Nhuan Hospital, Ho Chi Minh City, Vietnam; 2 HOPE Research Center, My Duc Hospital, Ho Chi Minh City, Vietnam; 3 Department of Obstetrics and Gynecology, My Duc Hospital, Ho Chi Minh City, Vietnam; 4 National Perinatal Epidemiology and Statistics Unit, Centre for Big Data Research in Health, University of New South Wales, Sydney, Australia; 5 Department of Obstetrics and Gynecology, Monash University, Clayton, Australia; 6 Department of Obstetrics and Gynaecology, Amsterdam UMC, University of Amsterdam, Amsterdam, the Netherlands; 7 Department of Obstetrics and Gynecology, University of Medicine and Pharmacy at Ho Chi Minh City, Ho Chi Minh City, Vietnam; King’s College, UNITED KINGDOM OF GREAT BRITAIN AND NORTHERN IRELAND

## Abstract

**Background:**

Pregnant women with twins and a short cervical length (CL) are at greater risk of preterm birth (PTB). The comparative efficacy of cervical cerclage and cervical pessary with or without additional progesterone to prevent PTB is unknown. We aimed to assess, in women with twin pregnancies and a short CL, the effectiveness of cerclage versus pessary and the additional treatment with 400 mg vaginal progesterone versus no progesterone in preventing PTB.

**Methods and findings:**

This multicenter, two-by-two factorial randomised trial was conducted in 2 hospitals in Ho Chi Minh City, Vietnam. Asymptomatic women with twin pregnancies and a CL ≤28 mm at 16 to 22 gestational weeks were recruited. Between March 2019 and July 2023, we randomised 219 participants (64.4% of the planned sample size) to cerclage plus progesterone (*n* = 55), Arabin pessary plus progesterone (*n* = 56), cerclage alone (*n* = 54) or Arabin pessary alone (*n* = 54). Primary outcome was any PTB <34 weeks. Following the second interim analysis, the study was terminated due to significantly lower rates of perinatal deaths and deliveries <28 weeks in the cerclage group. The primary outcome occurred in 20 (19.8%) participants receiving cerclage versus 20 (19%) participants receiving pessary (relative risk [RR] 1.04; 95% confidence interval [CI], 0.60 to 1.8). Delivery <28 weeks occurred in 1% versus 8.6% (RR 0.12; 95% CI, 0.01 to 0.52) and perinatal death occurred in 1% versus 5.8% (RR 0.17; 95% CI, 0.05 to 0.62) in the cerclage group and the pessary group, respectively. However, PTB <24 weeks, <32 weeks, and other neonatal outcomes were not significantly different between the 2 groups. For maternal side effects, vaginal discharge was significantly less frequent in the cerclage group. In participants allocated to progesterone, PTB <34 weeks occurred in 19 (18.4%) versus 21 (20.4%) participants who did not have progesterone (RR 0.90; 95% CI, 0.52 to 1.6).

**Conclusions:**

In this prematurely halted study on pregnant women with twins and a CL ≤28 mm, cerclage and cervical pessary were comparably effective on PTB <34 weeks prevention. However, compared to pessary, cerclage was associated with significantly lower rates of PTB <28 weeks and perinatal mortality.

ClinicalTrials.gov Registration: NCT03863613 (https://clinicaltrials.gov/study/NCT03863613)

## Abstract 2

In this randomized clinical trial, Yen TN He and colleagues compare the use of cerclage versus pessary with or without progesterone in the prevention of pre-term birth in twin pregnancies.

## Author summary

**Why was this study done?:**

- Women with twin pregnancies and a short cervix are at extremely high risk of preterm birth, the most common cause of newborn death and hospital readmission worldwide.
- Cervical cerclage, cervical pessary, and vaginal progesterone have been proposed as strategies to prevent or at least delay preterm birth, with controversial findings.
- Currently, there are no randomised trials directly comparing the effectiveness of cerclage versus pessary and evaluating the additional effect of progesterone in women with twin pregnancies and a short cervix.

**What did the researchers do and find?:**

- In this multicenter, two-by-two factorial, randomised trial, women with twin pregnancies and a cervix ≤28 mm were randomised to receive cervical cerclage with vaginal progesterone, Arabin pessary with vaginal progesterone, cervical cerclage alone, or Arabin pessary alone. The trial was stopped prematurely, due to safety concerns as the preterm birth <28 weeks and perinatal death rates were significantly lower in the cerclage group.
- Data from 219 (64.4% of the planned sample size) women was analysed. Women undergoing cerclage and pessary had a similar rate of preterm birth <34 weeks. However, the use of cerclage was associated with significantly lower rates of preterm birth <28 weeks and perinatal death. The addition of 400 mg vaginal progesterone to cerclage or pessary did not show significant additional beneficial effect.

**What do these findings mean?:**

- For women with twin pregnancies and a short cervix, cervical cerclage might be the method of choice to prevent preterm birth. The addition of vaginal progesterone to cerclage or pessary might have no additional beneficial effect.
- Further trials with adequate sample size and robust design are needed.

## Introduction

Preterm birth (PTB) is the most common cause of neonatal morbidity and mortality worldwide [1,2] and the leading cause of death in children under 5 years [3]. Women with twin pregnancies are at increased risk of PTB, as more than half of them deliver before 37 weeks, and 20% even before 34 weeks [4]. In addition, short cervical length (CL) in the second trimester of pregnancy is an independent predictor of PTB [5,6]. Therefore, women with twin pregnancies and a short cervix are at extremely high risk of PTB [7]. It has been suggested that universal cervical screening should be offered to all twin pregnancies [8]. For those with a short CL, vaginal progesterone, cerclage, and cervical pessary have been proposed as strategies to prevent or at least delay PTB [9].

In singleton pregnant individual with a short cervix, evidence from randomised clinical trials (RCTs) suggests that progesterone significantly reduces PTB and neonatal complications, while in those with multiple pregnancies, this effect is not supported by current evidence [10]. However, a recent RCT suggested that progesterone may reduce spontaneous birth <32 weeks in women with twin pregnancies and a CL <30 mm [11]. It is unclear if progesterone has any additional effect in those already treated with cerclage or pessary.

Pregnant women with short cervix, including singletons or twins, were suggested to be beneficial from pessary treatment by early RCTs [12–14]. However, other studies could not confirm these effects [15,16]. Studies directly comparing the efficacy of pessary and progesterone in women with singleton or twin pregnancies showed no clear benefit of one treatment over the other [17–19].

Evidence from RCTs on cerclage in women with twin pregnancies was limited. A meta-analysis using individual participant-level data in 49 women with twin pregnancies and a CL <25 mm showed that cerclage resulted in higher risks of early delivery, low birthweight, and neonatal respiratory distress syndrome [20]. In contrast, a recent meta-analysis including 2 RCTs and 13 cohort studies indicated that cerclage might be beneficial in twin pregnant women with a CL <15 mm [21]. We conducted this study to directly compare the efficacy of cerclage to pessary, in women with twin pregnancies and a short cervix. We also evaluated whether the addition of progesterone was effective.

## Methods

### Study design

This was an open-label, multicenter, two-by-two factorial RCT, performed at My Duc Hospital and My Duc Phu Nhuan Hospital, Ho Chi Minh City, Vietnam. Pregnant women were screened for eligibility between March 2019 and July 2023, with the last randomisation in January 2023; follow-up was completed in July 2023. The trial was conducted according to Good Clinical Practice and Declaration of Helsinki 2002 principles and had oversight provided by an independent Data Safety Monitoring Committee (DSMC). The study protocol was approved by the Institutional Ethics Committee of My Duc Hospital (02/2019/MĐ-HĐĐĐ), registered in ClinicalTrials.gov (NCT03863613) and published previously [22]. All participants provided written informed consent prior to randomisation. This study collected data on biological sex but not gender identity. Where the words women, she or her are used, it is to describe individuals whose sex was assigned at birth as female, whether they identify as female, male, or binary.

### Participants

Asymptomatic pregnant women with twins, irrespective of chorionicity, were considered for this trial. In our previous study [18], the 25th percentile cut-off of cervical length in women with twins was ≤28 mm. Eligibility criteria included a transvaginal CL ≤28mm at 16 to 22 weeks’ gestation. The following variables were exclusion criteria: uterine anomalies; cervical dilation with visible amniotic membranes or amniotic membranes prolapsed into the vagina; twin-to-twin transfusion syndrome; stillbirth of one of the twins; major congenital abnormalities in any of the fetuses; severe vaginal discharge; acute vaginitis or cervicitis; vaginal bleeding, placenta preavia or vasa preavia; premature rupture of membranes or premature labor with/without ruptured membrane; suspicion of chorioamnionitis; cerclage or pessary in place or unable to undergo cerclage or pessary placement.

### Procedures

All women with twin pregnancies, at 16^0/7^ to 22^0/7^ weeks’ gestation, underwent CL measurement and digital examination routinely. For women conceived through assisted reproductive technology (ART), gestational age was determined by the date of embryo transfer or intrauterine insemination. For those conceived naturally, gestational age was calculated based on the last menstrual period and confirmed by the fetal crown-rump length of the largest twin at first trimester ultrasound. Prior to CL measurement, they were given a brochure outlining risk factors and available PTB prevention methods. CL measurement was performed by 4 ultrasonographers (B.H.V., M.T.L., A.P.T.P., and H.H.N.P.), 2 in each hospital, who were all certificated by the Fetal Medicine Foundation. Women with a CL ≤28 mm were invited to participate in the study and were provided written information. After a subsequent discussion with investigators, if the women agreed to participate, they were asked to sign the consent form prior to study enrollment.

### Randomisation and masking

Randomisation was carried out by entering participant details into a web portal of HOPE Research Center, My Duc Hospital. Treatment allocation was then assigned according to a computer-generated randomisation list stored in the online system. Participants were randomly allocated, in a 1:1:1:1 ratio, to cerclage with progesterone, Arabin pessary with progesterone, cerclage alone, or Arabin pessary alone, using block randomisation with a variable block size of 4 or 8. Due to the nature of interventions, only neonatologists assessing the neonates were unaware of treatment allocation.

### Interventions

In participants allocated to cerclage, a Mersilene suture (Ethicon, LLC, United States) was placed around the endocervical canal using McDonald technique under spinal anesthesia. Intravenous prophylactic antibiotics, Cefazolin (Zoliicef 1g, Pymepharco, Vietnam), were given 1 h before the procedure. For women reporting an allergy to cephalosporin, Clindamycin (Clindamycin Hameln 300 mg 2 ml, Siegfried Hameln GmBH, Germany) or Vancomycin (Vancomycin hydrochloride 1g, Xellia Pharmaceuticals ApS, Denmark) was used [23].

In participants allocated to pessary, a soft, flexible, silicone cervical pessary (Arabin, Dr Arabin GmbH & Co KG, Germany) was inserted through the vagina, upward around the cervix. The pessary size was based on digital examination, with the initial size guidance from the publication by Arabin and Alfirevic [24]. Four senior clinicians (Y.T.N.H., C.H.L., T.Q.B., and N.T.V.; 2 in each hospital), who each had at least 5 years of experience with cervical cerclage and pessary placement, were involved in the procedures. The interventions were intended to be performed within 7 days after randomisation.

In participants allocated to additional progesterone, 400 mg vaginal progesterone was applied (Cyclogest 400 mg, Actavis, United Kingdom), once daily at bedtime, starting from the day of receiving cerclage or pessary. Participants were required to record their drug use in a diary sheet for up to 147 days. At every visit, their compliance was documented by checking the diary and drug purchasing records from the hospital pharmacy. Compliance rate was calculated by dividing the number of progesterone doses used by the number of progesterone doses that should have been used since the last visit. Participants were considered compliant when their drugs used-to-prescribed rate was ≥80%.

Follow-up examination was at 14 days post-randomisation, and then weekly or monthly according to the pregnancy stage and the presence of complaints. At every visit, participants underwent routine care to reveal any adverse events or complications. CL measurement was not performed routinely after randomisation, unless on the participant’s request. In case of premature rupture of the membranes, active vaginal bleeding, other signs of preterm labor, or severe discomfort of the participant, the use of pessary, cerclage, and/or progesterone was discontinued. Further treatment was indicated per local protocol. All interventions were terminated at 37 weeks or at delivery, whichever came first. For method of delivery, it was our practice to offer elective cesarean section (C-section) to pregnant women with twin pregnancies at a gestational age of ≥37 weeks or in case of premature rupture of membranes [25,26]. The choice for a C-section or vaginal delivery was left to the pregnant women and their partners.

### Outcomes

The primary outcome was any PTB <34 weeks’ gestation. Secondary outcomes consisted of fetal death <24 weeks, stillbirth ≥28 weeks, live birth, delivery mode, gestational age at delivery, PTB <24, <28, <32, and <37 weeks, spontaneous and iatrogenic PTB <28, <34, and <37 weeks, time from randomisation to delivery, labor induction, tocolytic drugs, antenatal corticosteroids and magnesium sulfate for neuroprotection use, admission days for preterm labor, preterm premature rupture of membrane, chorioamnionitis, maternal side effects (vaginal discharge, fever, vaginal infection or pain, pessary repositioning, and necrosis or rupture of the cervix), maternal morbidity (thromboembolic complications, urinary tract infection treated with antibiotics, pneumonia, endometritis, hypertensive disorder, eclampsia, hemolysis, elevated liver enzymes, low platelet count syndrome, death). Neonatal outcomes included birthweight, birthweight <1,500 g and <2,500 g, congenital anomalies diagnosed after randomisation, 5-min Apgar score, 5-min Apgar score <7, perinatal death, death before discharge, neonatal intensive care unit (NICU) admission, admission days to the NICU, intraventricular hemorrhage, respiratory distress syndrome, necrotizing enterocolitis, proven sepsis, and a composite of poor perinatal outcomes. Stillbirth <28 and <34 weeks were added post hoc as secondary endpoints. Definitions of all endpoints are provided in S12 Table.

### Safety

Participants, after randomisation, continued receiving primary care as usual and were advised to contact the research team or their treating physicians whenever they needed any additional information or support from healthcare professionals. All adverse events were documented and reported in line with the institutional review board’s (IRB) guidelines and were followed until the participants’ situation was stable.

### Sample size

We would like to evaluate whether the use of cerclage would reduce the relative risk of PTB rate at <34 weeks by 50%. Using preliminary data from our previous study [18], we determined that a sample size of 320 participants would be required to show that cerclage would decrease the PTB rate from 24% to 12%, with an alpha-level of 0·05, power of 80%. This number would also give us 80% power to show or refute a reduction in PTB in the progesterone group, from 55% to 39%. We did not consider an interaction between the cerclage/pessary and additional progesterone/no progesterone comparisons. Considering a 5% lost to follow-up and protocol deviation, our goal was to recruit a total of 340 participants (85 per arm).

### Statistical analysis

Data analysis was performed on an intention-to-treat basis. In view of the two-by-two factorial design, the analysis was done separately for the cerclage versus pessary and for the additional progesterone versus no progesterone comparison. For categorical variables, summary data were reported as proportions. For continuous variables, results were reported as a mean and standard deviation for normally distributed variables, or median and interquartile range (Q1; Q3) for non-normally distributed variables. Between-group differences were assessed using Student *T* test or Mann–Whitney U test for continuous outcomes and Chi-squared or the Fisher exact test for categorical outcomes.

Relative risk (RR) and 95% confidence interval (CI) intervals were calculated for dichotomous endpoints. For time-to-delivery, we estimated Kaplan–Meier curves and performed Cox proportional hazard analysis, where the gestational week at delivery was the time scale, and delivery was the event. Hazard ratio (HR) values were estimated using a Cox proportional hazards model, with a formal test of the proportional hazard assumption. For neonatal dichotomous outcomes, we utilised cluster analysis with the generalised estimating equations (GEE) model using the Poisson family, robust estimate for standard error and an exchangeable within-group correlation structure to account for the twins’ dependency and estimate the 95% CI [27]. For maternal outcomes, the Wald method was used to calculate RR and 95% CI intervals for dichotomous endpoints.

For participants with no data on the primary outcome, we first performed analysis by excluding missing values and then explored a best and worst case scenario in both groups, assuming all participants lost to follow up had either a PTB <34 weeks (worst case) or a birth at ≥34 weeks’ gestation (best case). For those with missing values related to secondary outcomes, data analysis was performed by excluding missing values. We planned a prespecified subgroup analysis by quartiles of CL. We tested for interaction between CL and the treatment effect on PTB <34 weeks, the composite of poor perinatal outcomes, and perinatal death. The exact test with mid-P method was used [28], with *p*-values <0.05 being considered to indicate statistically significant. As per statistical analysis plan, we also performed a per-protocol analysis. Statistical analyzes were performed using R statistical software, version 4.3.0.

We planned an interim analysis after completion of data collection of 150 randomised participants, using a two-sided significance test with the Haybittle–Peto spending function and a type I error rate of 5% with stopping criteria of *p* < 0.001 (Z-alpha = 3.29) and assessing data for safety, efficacy, and futility. At the first interim analysis in October 2021, which included complete data of 156 participants, the DSMC recommended continuing recruitment till complete assemblage of two thirds of the total sample’s data to undergo a second interim analysis. The second interim analysis was performed in July 2023 and included complete data of 219 (64.4% of the planned sample size) participants.

## Results

### Participant disposition

**Fig 1:**
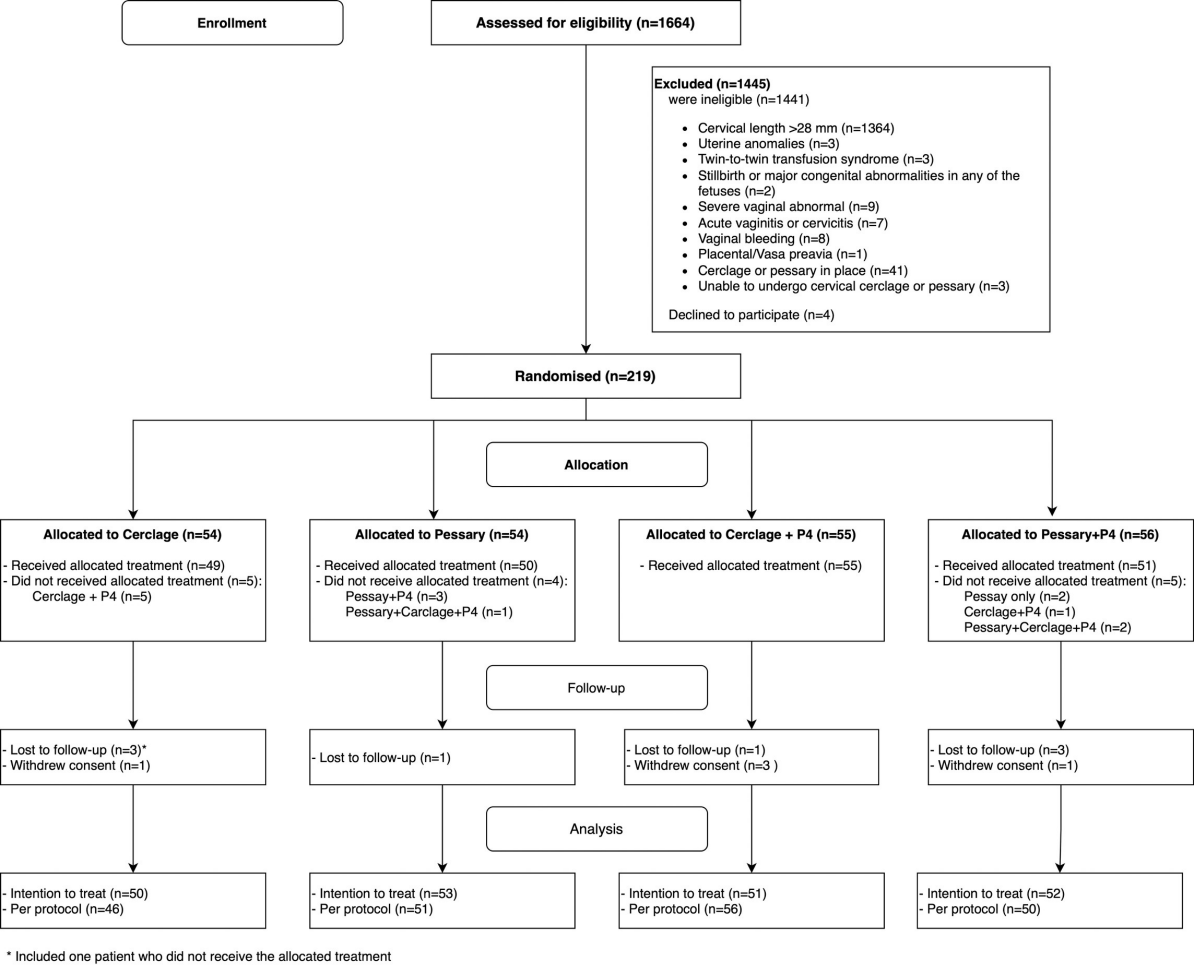
Trial profile.

Between 22 March 2019 and 29 July 2023, we screened 1,664 pregnant women, of whom 223 met the eligibility criteria (Fig 1). Four women declined to participate. Therefore, 219 participants were randomised to cerclage with progesterone (*n* = 55), pessary with progesterone (*n* = 56), cerclage alone (*n* = 54), or pessary alone (*n* = 54). Due to safety concerns as the PTB <28 weeks and perinatal death rates were significantly higher in the pessary group compared to cerclage (8.6% versus 1% and 8.6% versus 2%, respectively), following the DSMC’s recommendation, the trial was stopped on 29 July 2023. There were 201 participants with a dichorionic twin pregnancy (91.8%) and 18 with a monochorionic diamniotic twin pregnancy (8.2%) while none had a monoamniotic twin pregnancy. Baseline characteristics of participants were comparable between the 2 groups (Table 1).

**Table 1:** Baseline characteristics.

	All (*N* = 219)	Cerclage vs. Pessary		Progesterone vs. No Progesterone	
		Cerclage (*N* = 109)	Pessary (*N* = 110)	Progesterone (*N* = 111)	No Progesterone (*N* = 108)
Maternal age, mean (SD), y	31.3 (4.6)	31.3 (4.4)	31.2 (4.8)	31.0 (4.4)	31.6 (4.8)
Body mass index, median (Q1;Q3), kg/m^2^	21.2 (19.6;22.8)	21.0 (19.4;22.8)	21.4 (19.7;22.9)	21.3 (19.6;22.7)	21.5 (19.7;23.3)
Number of previous pregnancy, No. (%)					
Nulliparous	127 (58)	59 (54.1)	68 (61.8)	62 (55.9)	65 (60.2)
1	63 (28.8)	31 (28.4)	32 (29.1)	33 (29.7)	30 (27.8)
≥ 2	29 (13.2)	19 (17.5)	10 (9.1)	16 (14.4)	13 (12)
Prior miscarriage, No. (%)	54 (24.7)	26 (23.9)	28 (25.5)	27 (24.3)	27 (25)
Prior preterm birth, No. (%)	1 (0.5)	0 (0)	1 (0.9)	0 (0)	1 (0.9)
Prior cervical surgery, No. (%)	3 (1.4)	0 (0)	3 (2.7)	2 (1.8)	1 (0.9)
Prior uterine surgery, No. (%)	26 (11.9)	17 (15.6)	9 (8.2)	15 (13.5)	11 (10.2)
Chorionicity, No. (%)					
Dichorinonic diamniotic	201 (91.8)	101 (92.7)	100 (90.9)	105 (94.6)	96 (88.9)
Monochorionic diamniotic	18 (8.2)	8 (7.3)	10 (9.1)	6 (5.4)	12 (11.1)
Conception, No. (%)					
Spontaneous	12 (5.5)	6 (5.5)	6 (5.5)	7 (6.3)	5 (4.6)
Ovulation induction/artificial insemination	17 (7.8)	7 (6.4)	10 (9.1)	11 (9.9)	6 (5.6)
In vitro fertilisation	190 (86.8)	96 (88.1)	94 (85.4)	93 (83.8)	97 (89.8)
Gestational age at randomisation, mean (SD), wk	18.1 (1.9)	18.1 (1.9)	18.1 (1.9)	18.2 (1.9)	18.0 (1.9)
Cervical length at randomisation, mean (SD), mm	25.8 (3.1)	25.5 (3.4)	26.0 (2.7)	25.9 (3.1)	25.7 (3.1)
Cervical length range, No. (%)					
13–24 mm	42 (19.2)	23 (21.1)	19 (17.3)	22 (19.8)	20 (18.5)
25–26 mm	46 (21)	23 (21.1)	23 (20.9)	19 (17.1)	27 (25)
27 mm	58 (26.5)	28 (25.7)	30 (27.3)	27 (24.3)	31 (28·7)
28 mm	73 (33.3)	35 (32.1)	38 (34.5)	43 (38.7)	30 (27·8)

In all participants, the interventions were performed within 2 days after randomisation. After randomisation, 14 participants did not receive the allocated treatment (5 in the cerclage group, 4 in the pessary group, and 5 in the pessary with progesterone group) (Fig 1). In participants treated with additional progesterone, the compliance rate was 101/103 (98%). Thirteen participants were not included in the main analysis due to being lost to follow-up (*n* = 8) or withdrawal of informed consent (*n* = 5) (Fig 1). Details of these participants are presented in S1 and S2 Tables.

### Primary outcome

The primary outcome, any PTB <34 weeks, occurred in 20 (19.8%) participants in the cerclage group versus in 20 (19%) of those in the pessary group (RR 1.04; 95% CI, 0.60 to 1.8). For the progesterone comparison, it occurred in 19 (18.4%) participants treated with additional progesterone versus in 21 (20.4%) participants without progesterone (RR 0.90; 95% CI, 0.52 to 1.6) (Table 2).

**Table 2:** Outcomes on maternal level (intention-to-treat).

	All (*N* = 206)	Cerclage vs. Pessary				Progesterone vs. No Progesterone			
		Cerclage (*N* = 101)	Pessary (*N* = 105)	Relative Risk (95% CI)	*p*-values	Progesterone (*N* = 103)	No Progesterone (*N* = 103)	Relative Risk (95% CI)	*p*-values
**Primary outcome**									
Preterm birth <34 wk, No. (%)	40 (19.4)	20 (19.8)	20 (19)	1.04 (0.60, 1.8)	0.892	19 (18.4)	21 (20.4)	0.90 (0.52, 1.58)	0.729
**Secondary outcome**									
Stillbirth ≥28 wk, No. (%)	1 (0.5)	0 (0)	1 (1)	-	-	0 (0)	1 (1)	-	-
Stillbirth <28 wk, No. (%)^a^	8 (3.9)	1 (1)	7 (6.7)	0.15 (0, 0.69)	0.041	5 (4.9)	3 (2.9)	1.26 (0.31, 5.10)	0.500
Stillbirth <34 wk, No. (%)^a^	9 (4.4)	1 (1)	8 (7.6)	0.13 (0, 0.62)	0.022	5 (4.9)	4 (3.9)	1.25 (0.25, 7.00)	0.749
Neonatal death <24 wk, No. (%)	2 (1)	0 (0)	2 (1.9)	-	-	2 (1.9)	0 (0)	-	-
Perinatal death, No. (%)^b^	11 (5.3)	2 (2)	9 (8.6)	0.23 (0, 0.83)	0.040	5 (4.9)	6 (5.8)	0.83 (0.17, 3.0)	0.769
Preterm birth <24 wk, No. (%)	3 (1.5)	0 (0)	3 (2.9)	-	-	3 (2.9)	0 (0)	-	-
Preterm birth <28 wk, No. (%)	10 (4.9)	1 (1)	9 (8.6)	0.12 (0, 0.52)	0.012	6 (5.8)	4 (3.9)	1.50 (0.40, 10.0)	0.540
Preterm birth <32 wk, No. (%)	21 (10.2)	9 (8.9)	12 (11.4)	0.78 (0.34, 1.77)	0.562	14 (13.6)	7 (6.8)	2.0 (0.84, 4.75)	0.115
Preterm birth <37 wk, No. (%)	127 (61.7)	66 (65.3)	61 (58.1)	1.12 (0.91, 1.40)	0.290	63 (61.2)	64 (62.1)	0.98 (0.79, 1.22)	0.887
Onset of labor, No. (%)^c^	100 (48.8)	51 (50.5)	49 (47.1)	1.07 (0.81, 1.42)	0.632	57 (55.9)	43 (41.7)	1.34 (1.01, 1.78)	0.045
Mode of delivery, No. (%)^c^									
C-section	195 (95.1)	99 (98)	96 (92.3)	1.07 (1.0, 1.14)	0.040	98 (96.1)	97 (94.2)	1.01 (0.95, 1.1)	0.769
Elective	105 (53.8)	52 (52.5)	53 (55.2)	-	0.305^d^	47 (48)	58 (59.8)	-	0.068^d^
Nonprogressive labor	88 (45.1)	47 (47.5)	41 (42.7)	-	-	51 (52)	37 (38.1)	-	-
Suspected fetal distress	2 (1)	0 (0)	2 (2.1)	-	-	0 (0)	2 (2.1)	-	-
Gestational age at delivery, mean (SD), wkc	35.3 (3.2)	35.5 (2.4)	35.0 (3.8)	-	0.217^e^	35.0 (3.6)	35.5 (2.7)	-	0.293^e^
Time from randomisation to delivery, median (Q1;Q3), d^c^	127 (111;138)	127 (111;138)	127 (110;139)	-	0.927^f^	127 (111;137)	128 (112;140)	-	0.421^f^

^a^ Post hoc analysis.

^b^ Defined as any stillbirth ≥20 weeks and neonatal death ≥20 weeks.

^c^ One case miscarriage excluded. *p*-values according to a dichotomous outcome were calculated using the Wald test.

^d^ *p*-values were calculated using the Chi-squared test.

^e^ *p*-values were calculated using the *T* test.

^f^ *p*-values were calculated using the Mann–Whitney U test.

### Secondary outcomes

**Fig 2:**
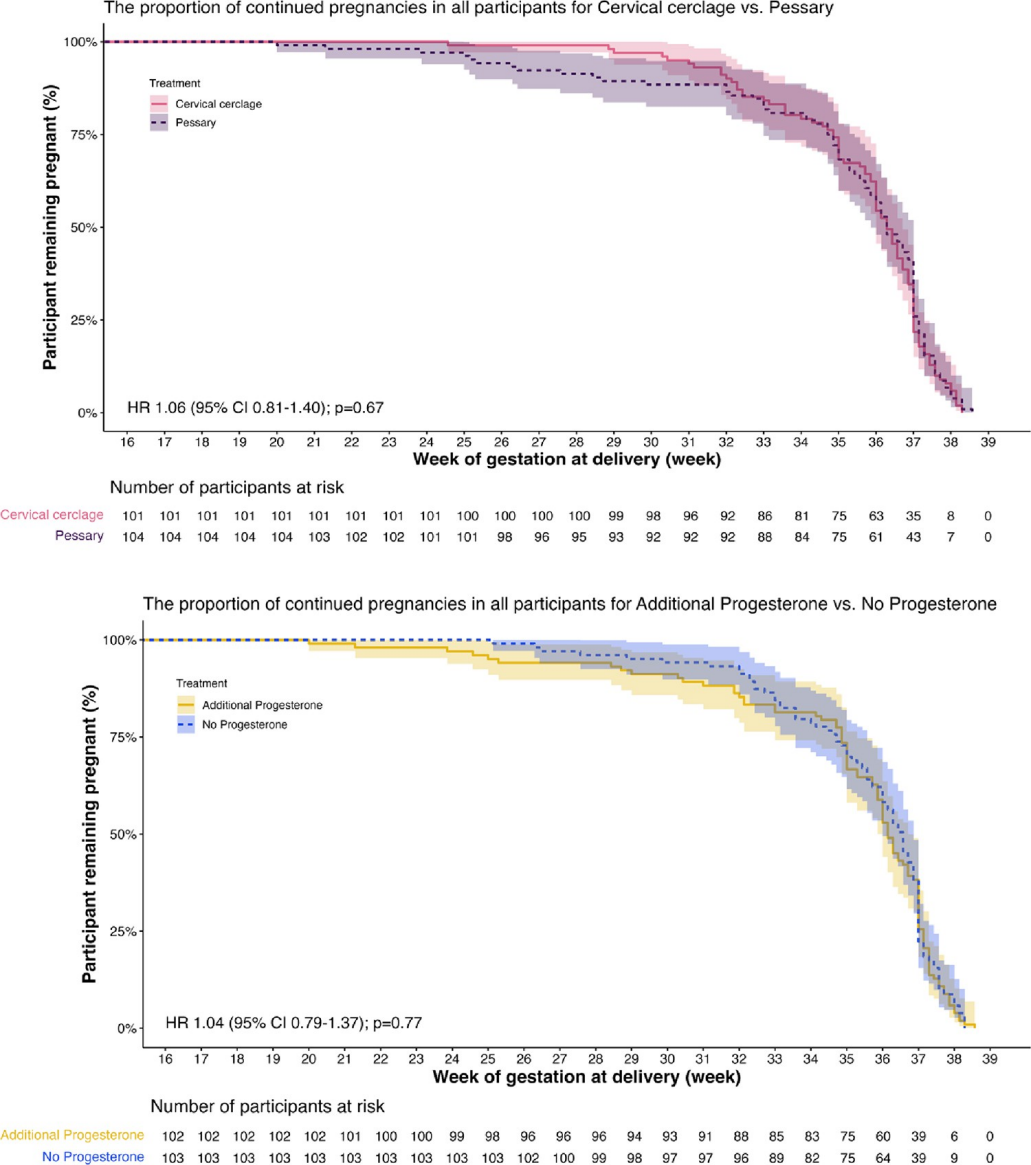
Kaplan–Meier curves showing the proportion of continued pregnancies in groups. (A) Participants treated with cerclage versus pessary (hazard ratio, 1.06; 95% CI, 0.81 to 1.4). (B) Participants treated with additional progesterone versus no progesterone (hazard ratio, 1.04; 95% CI, 0.79 to 1.4).

The time to delivery was not significantly different between participants in the cerclage group versus those in the pessary group (HR, 1.06; 95% CI, 0.81 to 1.4), as well as in participants treated with additional progesterone versus those without progesterone (HR, 1.04; 95% CI, 0.79 to 1.4) (Fig 2). Participants in the cerclage group had a significantly lower PTB <28 weeks rate compared to those in the pessary group (1% versus 8.6%, RR 0.12; 95% CI, 0 to 0.52). On maternal level, perinatal death was also significantly lower in participants treated with cerclage (Table 2). There were more participants in the cerclage group that had nonprogressive labor than those in the pessary group, but the difference was not statistically significant (53.4% versus 46.6%, *p* = 0.59), in S3 Table). However, in total, cesarean section was significantly more common in the cerclage group (Table 2). Other maternal outcomes and side effects are presented in S4 Table. There was 1 participant with pessary dispositioning, who felt pain and had constipation. The pessary was then removed and replaced. Data on maternal side effects are provided in S5 Table.

Analysis on neonatal level showed that compared to pessary, the use of cerclage was associated with significantly lower perinatal death (1% versus 5.8%, RR 0.17; 95% CI, 0.05 to 0.62) (Table 3). Birthweight <1,500 g occurred less often in the cerclage group compared to the pessary group (6.4% versus 12.6%, RR 0.51; 95% CI, 0.25 to 1.1), although this difference was not statistically significant. Other neonatal outcomes were not significantly different in the cerclage versus pessary and the addition of progesterone versus no progesterone comparisons (Table 3).

**Table 3:** Outcomes on neonatal level (intention-to-treat).

	All (*N* = 410)	Cerclage vs. Pessary				Progesterone vs No Progesterone			
Perinatal Outcomes		Cerclage (*N* = 202)	Pessary (*N* = 208)	Relative Risk (95% CI)	*p*-value	Progesterone (*N* = 204)	No Progesterone (*N* = 206)	Relative Risk (95% CI)	*p*-value
Stillbirth ≥28 wk, No. (%)	1 (0.2)	0 (0)	1 (0.5)	-	-	0 (0)	1 (0.5)	-	-
Stillbirth <28 wk, No. (%)^a^	9 (2.2)	1 (0.5)	8 (3.8)	0.13 (0.02, 0.74)	0.055	6 (2.9)	3 (1.5)	2.02 (0.61, 6.73)	0.337
Stillbirth <34 wk, No. (%)^a^	10 (2.4)	1 (0.5)	9 (4.3)	0.11 (0.02, 0.65)	0.040	6 (2.9)	4 (1.9)	1.51 (0.50, 4.58)	0.537
Neonatal death <24 wk, No. (%)	2 (0.5)	0 (0)	2 (1)	-	-	2 (1)	0 (0)	-	-
Perinatal death, No (%)^b^	14 (3.4)	2 (1)	12 (5.8)	0.17 (0.05, 0.62)	0.024	8 (3.9)	6 (2.9)	1.35 (0.50, 3.64)	0.622
Birth weight, mean (SD), g^c^	2,248 (577)	2,283 (498)	2,214 (645)	-	0.230^f^	2,231 (628)	2,265 (523)	-	0.546^f^
Birth weight <1,500 g, No. (%)^c^	39 (9.5)	13 (6.4)	26 (12.6)	0.51 (0.25, 1.07)	0.137	24 (11.8)	15 (7.3)	1.62 (0.81, 3.21)	0.251
Birth weight <2,500 g, No. (%)^c^	243 (59.4)	126 (62.4)	117 (56.5)	1.1 (0.94, 1.30)	0.294	125 (61.3)	118 (57.6)	1.07 (0.91, 1.26)	0.494
5-min Apgar score, median (Q1;Q3)^d^	9 (8;9)	9 (8;9)	9 (8;9)	-	0.355^g^	9 (8;9)	9 (8.2;9)	-	0.202^g^
Apgar score <7, No. (%)^d^	8 (3.1)	1 (0.8)	7 (5.5)	0.15 (0.02, 0.92)	0.086	4 (3.3)	4 (2.9)	1.01 (0.22, 4.62)	0.991
Congenital anomalies after randomisation, No. (%)	8 (2)	7 (3.5)	1 (0.5)	7.21 (1.18, 43.85)	0.072	1 (0.5)	7 (3·4)	0.14 (0.02, 0.88)	0.077
Admission to NICU, No. (%)	140 (34.1)	74 (36.6)	66 (31.7)	1.15 (0.85, 1.57)	0.438	64 (31.4)	76 (36.9)	0.85 (0.63, 1.16)	0.384
Length of NICU admission, median (Q1;Q3), d^e^	3 (2;10)	3 (2;12.8)	2 (2;9)	-	0.126^g^	3 (2;9.8)	3 (2;12)	-	0.717^g^
Respiratory distress syndrome, No. (%)	90 (22)	52 (25.7)	38 (18.3)	1.41 (0.95, 2.10)	0.157	47 (23)	43 (20.9)	1.10 (0.74, 1.64)	0.683
Intraventricular hemorrhage, No. (%)	1 (0.2)	0 (0)	1 (0.5)	-	-	1 (0.5)	0 (0)	-	-
Necrotizing enterocolitis, No. (%)	11 (2.7)	8 (4)	3 (1.4)	2.75 (0.68, 11.13)	0.235	8 (3.9)	3 (1.5)	2.69 (0.66, 10.91)	0.244
Proven sepsis, No. (%)	38 (9.3)	22 (10.9)	16 (7.7)	1.42 (0.76, 2.65)	0.361	14 (6.9)	24 (11.7)	0.59 (0.31, 1.13)	0.184
Composite of poor perinatal outcomes, No. (%)	105 (25.6)	59 (29.2)	46 (22.1)	1.32 (0.93, 1.88)	0.194	52 (25.5)	53 (25.7)	0.99 (0.71, 1.41)	0.966

NICU, neonatal intensive care unit.

^a^ Post hoc analysis.

^b^ Defined as any stillbirth ≥20 weeks and neonatal death ≥20 weeks.

^c^ One case with missing data excluded.

^d^ One hundred fifty-four cases with missing data (out of 410 babies) excluded.

^e^ Six cases with missing data (out of 140 NICU admission) excluded; *p*-values according to a dichotomous outcome were calculated using GEE model.

^f^ *p*-values were calculated using the *T* test.

^g^ *p*-values were calculated using the Mann–Whitney U test.

### Exploratory outcomes

The 25^th^, 50^th^, and 75^th^ percentile cut-offs of CL were 24 mm, 26 mm, and 27 mm, respectively. There was no statistically significant interaction between CL and treatment effect on PTB <34 weeks, composite of poor perinatal outcomes or perinatal death. Prespecified subgroup analysis showed that PTB <34 weeks, perinatal death and composite of poor perinatal outcomes were comparable between groups in participants with different CL percentiles (S6 Table). In the best and worst case scenario analysis for the primary outcome, we also found that any PTB <34 weeks was comparable between the cerclage group and the pessary group as well as between participants treated with additional progesterone and participants without progesterone (S7 Table). Results of per-protocol analyses of the primary outcome and the rates of stillbirth, perinatal death were similar to those of the intention-to-treat analyses (S8–S10 Tables).

## Discussion

In this prematurely halted multicenter, two-by-two factorial RCT, we found that the rate of PTB <34 weeks’ gestation was comparable between cerclage and cervical pessary. However, cerclage was associated with significantly lower rates of PTB <28 weeks and perinatal death. The addition of 400 mg vaginal progesterone to cerclage or pessary did not show significant additional beneficial effect. There was no statistically significant interaction between CL and treatment effect on PTB <34 weeks, composite of poor perinatal outcomes or perinatal death.

Strengths of our trial include the prespecified and published protocol [22], as well as limited number of accredited professionals involving in CL measurement and cerclage and pessary application. All involved clinicians had at least 5 years of experience with the procedures. Moreover, the two-by-two factorial design allowed us to make 2 comparisons at once.

Our trial also has limitations. First, the study had an open design due to the nature of the interventions. Attempts to minimise bias included comparable and protocolized follow-up in both groups. Also, neonatologists assessing newborns were unaware of treatment allocation in order to prevent ascertainment bias. Second, most participants in the trial conceived through ART treatment were nulliparous and had low body mass index which might compromise the generalisability of our findings. Third, our trial was powered to detect 50% reduction in PTB <34 weeks, from 24% to 12%, between cerclage and pessary, and was not powered to detect a smaller difference. However, targeting for a smaller difference would make the sample size unfeasibly large since women with twin pregnancies and a short cervix present relatively infrequent, also due to the fact that the implementation of single embryo transfer policy in in vitro fertilisation (IVF) reduced the number of twins. Finally, the early stop of the trial before planned enrollment completed, based on the DSMC’s recommendation due to the safety concern of pessary use was associated with more stillbirths, limited the power to assess the efficacy of progesterone. As we have started to promote single embryo transfer since 2018, the number of twin pregnancies available from our IVF program has been dramatically decreased. Consequently, there were no eligible participants since 23 January 2023 (the date of last patient in). Last patient follow-up was completed on 08 July 2023.

For pessary, large RCTs performed a decade ago suggested that it may benefit women with twin pregnancies and a short cervix, although results were inconsistent [12,13,15]. Recent studies in women with singleton pregnancies and a short cervix have made clear that pessary did not reduce PTB sufficiently [29]. Our previous RCT suggested that pessary might be superior to 400 mg vaginal progesterone for prevention of PTB and improvement of outcomes in women with twin pregnancies and a CL <28 mm [18]. Findings from this current study demonstrated that cerclage might be better than pessary in preventing PTB <28 weeks and perinatal mortality in the same high-risk population. Therefore, among these 3 proposing methods, and subjective to confirmation in other studies, cerclage seems to be the most effective intervention.

In the current study, our data showed that compared to pessary, cerclage significantly decreased the perinatal death (1% versus 5.8%, RR 0.17; 95% CI, 0.05 to 0.62), which resulted mostly from stillbirths <28 weeks (0.5% versus 3.8%, RR 0.13; 95% CI 0.02 to 0.74, respectively). In line with our findings, a recent trial on women with singleton pregnancies and a CL ≤20 mm showed that fetal or neonatal/infant death, one of the secondary outcomes, occurred more frequently in those randomised to receive a pessary (13.3%) than those randomised to receive usual care (6.8%) (RR 1.94; 95% CI, 1.1 to 3.3) [29]. It should be noted that these findings have not been previously reported in women with twin pregnancies. One possible explanation could be the fact that, compared to our study, the previous trials used a higher cut-off CL [13,30,31] and/or applied intervention at a later gestational age [12,15,31]. In the current trial, we recruited participants with severe CL shortening found early in gestation, with mean gestational age at randomisation was 18 weeks. Furthermore, the quality of earlier studies reporting on cerclage in twins was generally considered to be of average to above average, with many randomised trials having small sample size, as indicated in the latest Cochrane review [32]. In addition, the PTB rates in the pessary group found in this current study were equal to the PTB rates observed in our previous study (PTB <28 weeks was 9% and PTB <34 weeks was 21%, in twins with CL ≤28 mm (25th percentile) in the pessary group) [18]. It is not indicated that the difference between cerclage and pessary was due to harm caused by the pessary. Nevertheless, other rationales for these findings need to be elucidated in future.

Findings from the current study may change the attitude to cerclage in women with twin pregnancies and a short CL. Cerclage has long been denied for this indication, as smaller RCTs suggested harm from cerclage in twins [20,33]. The primary finding of this study indicated that cerclage might be more effective than pessary. In line with our results, a recent meta-analysis of cohort studies also indicated benefit of cerclage in women with twin pregnancies and a short cervix [21]. In fact, no RCTs on preventive interventions have shown a reduction of PTB rate <28 weeks to 1% as cerclage did in our study (Tables 2 and S11) [12,13,34,35]. It might be argued that these findings could be due to chance. However, there was a clear gradient pattern, in favour of cerclage, for PTB <37, <34, <32, <28, and <24 weeks. Accordingly, the positive finding for PTB <28 weeks is unlikely to be due to the inflated type-I error. This pattern can also be observed from the Kaplan–Meier curves. We, however, acknowledged the statistically significant findings are secondary outcomes. Therefore, these findings need to be confirmed by further trials, and the exact CL cut-off for which cerclage is effective needs to be determined. The generally accepted cut-off value for CL as a predictor of PTB is 25 mm, a value that is derived from singleton pregnancies. Ideally, cut-off values should be derived from RCTs that indicate a treatment benefit in a particular patient group. In the current trial, we chose 28 mm as a cut-off based on a preplanned subgroup analysis of our previous RCT reporting that in women with a CL ≤25^th^ percentile (≤28 mm), the use of pessary was associated with a significant reduction in the risk of PTB <34 weeks, PTB <37 weeks and the risk of poor perinatal outcomes [18]. There is scarce evidence regarding the management of women with a CL between 25 and 28 mm, who are likely to have an elevated risk of PTB. Only by including women below a cut-off 28 mm, we would be able to understand how pessary compared to cerclage in women with a CL between 25 mm and 28 mm. In fact, in the current study, more than 80% of the participants were in women with CL between 25 and 28 mm, and there was a clear treatment benefit in these women.

Our data also suggested that the addition of 400 mg vaginal progesterone to cerclage or pessary had no significant additional beneficial effect on the prevention of PTB or other outcomes in women with twin pregnancies and a short cervix. This finding needs to be extrapolated with caution due to the premature termination of the trial and the small number of events in the progesterone analysis. While it is clear that progesterone is not effective in unselected women with twin pregnancies [10,11,36], in those with a CL <30 mm, a recent RCT suggested that early use of 600 mg progesterone may reduce the risk of spontaneous PTB <32 weeks [11]. Other trials on this population are ongoing (e.g., NCT05968794, NCT03340688).

In conclusion, in this prematurely halted two-by-two factorial study on women with twin pregnancies and a CL ≤28 mm, cerclage and cervical pessary were associated with comparable PTB rates <34 weeks’ gestation. However, PTB <28 weeks and perinatal death rates were significantly lower in the cerclage group compared to pessary. We did not find a significant additional preventative effect of vaginal progesterone to cerclage/pessary.

## Supporting information

S1 CONSORT Checklist CONSORT 2010 Checklist of Information to Include When Reporting a Randomised Trial^a^. (PDF)

S1 Table Data of 8 individuals lost to follow-up. (DOCX)

S2 Table Data of 5 individuals withdrawing informed consent. (DOCX)

S3 Table Reasons for undergoing cesarean section. (DOCX)

S4 Table Additional outcomes on maternal level (intention-to-treat). (DOCX)

S5 Table Maternal side effects. (DOCX)

S6 Table Treatment outcomes in different quartiles of cervical length (intention-to-treat analysis). (DOCX)

S7 Table Best and worst case scenarios of PTB <34 weeks of all randomised participants. (DOCX)

S8 Table Outcomes on maternal level (per-protocol analysis). (DOCX)

S9 Table Outcomes on neonatal level (per-protocol analysis). (DOCX)

S10 Table Treatment outcomes in different quartiles of cervical length (per-protocol analysis). (DOCX)

S11 Table PTB <28 weeks’ gestation in different quartiles of cervical length. (DOCX)

S12 Table Secondary outcomes’ definition. (DOCX)

S1 Protocol The effectiveness of cervical pessary compared to cervical cerclage with or without vaginal progesterone for the prevention of preterm birth in women with a twin pregnancy and a short cervix: A two-by-two factorial randomised clinical trial. (DOCX)

## Abbreviations

ART: assisted reproductive technology
CI: confidence interval
CL: cervical length
DSMC: Data Safety Monitoring Committee
GEE: generalised estimating equation
HR: hazard ratio
IRB: institutional review board
NICU: neonatal intensive care unit
PTB: preterm birth
RCT: randomised clinical trial
RR: relative risk
