# Supplementary material for: Cervical cerclage versus cervical pessary with or without vaginal progesterone for preterm birth prevention in twin pregnancies and a short cervix: A two-by-two factorial randomised clinical trial
Source: PLoS Med. 2025 Feb 21;22(2):e1004526. doi: 10.1371/journal.pmed.1004526 (PMC11844863; doi:10.1371/journal.pmed.1004526)
Supplement: S1 Table — (DOCX) [file pmed.1004526.s002.docx]

S1 Table: Data of eight individuals lost to follow-up

| **Women** | **Allocation** | **Cervical length at randomization** | **Gestational age at randomization** | **Gestational age at last visit** | **Maternal status at last visit** | **Fetal status at last visit** |
| --- | --- | --- | --- | --- | --- | --- |
| 1 | Cerclage | 26 | 16.0 | 32.8 | Normal | Normal |
| 2 | Cerclage | 25 | 16.4 | 30.2 | Normal | Normal |
| 3 | Cerclage | 25 | 17.1 | 33.7 | Normal | Normal |
| 4 | Cerclage+Progesterone | 27 | 19.1 | 22.1 | Normal | Normal |
| 5 | Pessary | 25 | 16.3 | 18.2 | Normal | Normal |
| 6 | Pessary+Progesterone | 28 | 16.4 | 22.4 | Normal | Normal |
| 7 | Pessary+Progesterone | 28 | 22.0 | 23.1 | Normal | Normal |
| 8 | Pessary+Progesterone | 25 | 21.1 | 30.1 | Normal | Normal |
