# Supplementary material for: Cervical cerclage versus cervical pessary with or without vaginal progesterone for preterm birth prevention in twin pregnancies and a short cervix: A two-by-two factorial randomised clinical trial
Source: PLoS Med. 2025 Feb 21;22(2):e1004526. doi: 10.1371/journal.pmed.1004526 (PMC11844863; doi:10.1371/journal.pmed.1004526)
Supplement: S2 Table — (DOCX) [file pmed.1004526.s003.docx]

S2 Table: Data of five individuals withdrawing informed consent

| **Women** | **Allocation** | **Cervical length at randomization** | **Gestational age at randomization** | **Gestational age at last visit** | **Maternal status at last visit** | **Fetal status at last visit** |
| --- | --- | --- | --- | --- | --- | --- |
| 1 | Cerclage | 28 | 19.6 | 20 | Normal | Normal |
| 2 | Cerclage+Progesterone | 13 | 21.0 | 28.3 | No information | No information |
| 3 | Cerclage+Progesterone | 22 | 17.0 | 17.4 | Normal | Normal |
| 4 | Cerclage+Progesterone | 22 | 18.9 | 19.3 | Normal | Normal |
| 5 | Pessary+Progesterone | 28 | 20.7 | 31.1 | No information | No information |
