# Supplementary material for: Cervical cerclage versus cervical pessary with or without vaginal progesterone for preterm birth prevention in twin pregnancies and a short cervix: A two-by-two factorial randomised clinical trial
Source: PLoS Med. 2025 Feb 21;22(2):e1004526. doi: 10.1371/journal.pmed.1004526 (PMC11844863; doi:10.1371/journal.pmed.1004526)
Supplement: S3 Table — (DOCX) [file pmed.1004526.s004.docx]

S3 Table: Reasons for undergoing Cesarean section

| C-section | Cervical cerclage | Pessary | p-value |
| --- | --- | --- | --- |
| Elective, No. (%) | 52/105 (49.5) | 53/105 (50.5) | 0.99 |
| Nonprogressive labor, No. (%) | 47/88 (53.4) | 41/88 (46.6) | 0.59 |
| Suspected fetal distress, No. (%) | 0/2 (0) | 2/2 (100) | 0.48 |
