# Supplementary material for: Cervical cerclage versus cervical pessary with or without vaginal progesterone for preterm birth prevention in twin pregnancies and a short cervix: A two-by-two factorial randomised clinical trial
Source: PLoS Med. 2025 Feb 21;22(2):e1004526. doi: 10.1371/journal.pmed.1004526 (PMC11844863; doi:10.1371/journal.pmed.1004526)
Supplement: S4 Table — (DOCX) [file pmed.1004526.s005.docx]

S4 Table: Additional outcomes on maternal level (intention-to-treat)

|  |  | Cerclage versus Pessary | | | | Progesterone versus No Progesterone | | | |
| --- | --- | --- | --- | --- | --- | --- | --- | --- | --- |
|  | All (N=206) | Cerclage (N=101) | Pessary (N=105) | Relative Risk  (95% CI) | p-values | Progesterone (N=103) | No Progesterone (N=103) | Relative Risk (95% CI) | p-values |
| Spontaneous preterm birth <28 weeks, No. (%) | 9 (4.4) | 1 (1.0) | 8 (7.6) | 0.13 (0-0.62) | 0.022 | 6 (5.8) | 3 (2.9) | 1.51 (0.39-5.89) | 0.334 |
| Spontaneous preterm birth <34 weeks, No. (%) | 38 (18.4) | 19 (18.8) | 19 (18.1) | 1.04 (0.59-1.85) | 0.895 | 19 (18.4) | 19 (18.4) | 1 (0.56-1.78) | 0.951 |
| Spontaneous preterm birth <37 weeks, No. (%) | 94 (45.6) | 52 (51.5) | 42 (40.0) | 1.29 (0.95-1.74) | 0.101 | 50 (48.5) | 44 (42.7) | 1.14 (0.84-1.53) | 0.406 |
| Iatrogenic preterm birth <28 weeks, No. (%) | 1 (0.5) | 0 (0.0) | 1 (1.0) | - | - | 0 (0.0) | 1 (1.0) | - | - |
| Iatrogenic preterm birth <34 weeks, No. (%) | 2 (1.0) | 1 (1.0) | 1 (1.0) | 0.52 (0.03-8.28) | 0.981 | 0 (0.0) | 2 (1.9) | - | - |
| Iatrogenic preterm birth <37 weeks, No. (%) | 33 (16.0) | 14 (13.9) | 19 (18.1) | 0.77 (0.41-1.44) | 0.416 | 13 (12.6) | 20 (19.4) | 0.65 (0.34-1.24) | 0.191 |
| Thromboembolic complications, No. (%) | 0 (0) | 0 (0) | 0 (0) | - | - | 0 (0) | 0 (0) | - | - |
| Urinary tract infections treated with antibiotics, No. (%) | 12 (5.8) | 6 (5.9) | 6 (5.7) | 1.04 (0.30-4.16) | 0.946 | 7 (6.8) | 5 (4.9) | 1.4 (0.40-6.00) | 0.570 |
| Pneumonia, No. (%) | 0 (0) | 0 (0) | 0 (0) | - | - | 0 (0) | 0 (0) | - | - |
| Endometritis, No. (%) | 0 (0) | 0 (0) | 0 (0) | - | - | 0 (0) | 0 (0) | - | - |
| Hypertensive disorder, No. (%) | 22 (10.7) | 10 (9.9) | 12 (11.4) | 0.87 (0.39-1.92) | 0.731 | 10 (9.7) | 12 (11.7) | 0.83 (0.38-1.84) | 0.661 |
| Eclampsia, No. (%) | 0 (0) | 0 (0) | 0 (0) | - | - | 0 (0) | 0 (0) | - | - |
| HELLP syndrome, No. (%) | 0 (0) | 0 (0) | 0 (0) | - | - | 0 (0) | 0 (0) | - | - |
| Gestational diabetes, No. (%) | 71 (34.5) | 36 (35.6) | 35 (33.3) | 1.07 (0.73-1.56) | 0.730 | 39 (37.9) | 32 (31.1) | 1.22 (0.83-1.78) | 0.310 |
| Use of antenatal corticosteroids, No. (%) | 193 (93.7) | 98 (97.0) | 95 (90.5) | 1.07 (1-1.15) | 0.059 | 98 (95.1) | 95 (92.2) | 1.03 (0.96-1.11) | 0.410 |
| Use of MgSO4 for neuroprotection, No. (%) | 10 (4.9) | 3 (3.0) | 7 (6.7) | 0.45 (0-1.56) | 0.240 | 6 (5.8) | 4 (3.9) | 1.5 (0.40-9.00) | 0.540 |
| Use of tocolytic drugs, No. (%) | 10 (4.9) | 5 (5.0) | 5 (4.8) | 1.04 (0.21-5.20) | 0.951 | 6 (5.8) | 4 (3.9) | 1.5 (0.38-9.00) | 0.540 |
| Admission days for preterm labor, median (Q1, Q3), d ^a^ | 6.(5.0-8.5) | 6 (5-7) | 6 (5-11) | - | 0.248^b^ | 6 (5-9) | 6 (5-8) | - | 0.736^b^ |
| Preterm prelabour rupture of membranes, No. (%) | 44 (21.4) | 23 (22.8) | 21 (20.0) | 1.14 (0.67-1.92) | 0.632 | 21 (20.4) | 23 (22.3) | 0.91 (0.54-1.54) | 0.738 |
| Chorioamnionitis, No. (%) | 0 (0) | 0 (0) | 0 (0) | - | - | 0 (0) | 0 (0) | - | - |
| Live birth, No. (%) | 204 (99.0) | 101 (100.0) | 103 (98.1) | 1.02 (0.99-1.05) | 0.259 | 101 (98.1) | 103 (100.0) | 0.98 (0.95-1.01) | 0.249 |

^a^ 115 cases admitted for preterm labor, *p*-values according to a dichotomous outcome were calculated using the Wald test, ^b^*p*-values were calculated using the Mann–Whitney U test
