# Supplementary material for: Cervical cerclage versus cervical pessary with or without vaginal progesterone for preterm birth prevention in twin pregnancies and a short cervix: A two-by-two factorial randomised clinical trial
Source: PLoS Med. 2025 Feb 21;22(2):e1004526. doi: 10.1371/journal.pmed.1004526 (PMC11844863; doi:10.1371/journal.pmed.1004526)
Supplement: S5 Table — (DOCX) [file pmed.1004526.s006.docx]

S5 Table: Maternal side effects

|  | Cerclage versus Pessary | | | | | Progesterone vs No Progesterone | | | |
| --- | --- | --- | --- | --- | --- | --- | --- | --- | --- |
|  | **All (N=206)** | **Cerclage (N=101)** | **Pessary (N=105)** | **Relative Risk  (95% CI)** | **p-values** | **Progesterone (N=103)** | **No Progesterone (N=103)** | **Relative Risk (95% CI)** | **p-values** |
| Vaginal infection, No. (%) | 3 (1.5) | 1 (1.0) | 2 (1.9) | 0.35 (0.03-3.8) | 0.645 | 3 (2.9) | 0 (0.0) | - | - |
| Vaginal discharge, No. (%) | 71 (34.5) | 21 (20.8) | 50 (47.6) | 0.44 (0.28-0.67) | <0.001 | 30 (29.1) | 41 (39.8) | 0.73 (0.5-1.07) | 0.110 |
| Vaginal pain, No. (%) | 39 (18.9) | 17 (16.8) | 22 (21.0) | 0.8 (0.45-1.42) | 0.458 | 20 (19.4) | 19 (18.4) | 1.05 (0.6-1.85) | 0.861 |
| Necrosis or rupture of the cervix, No. (%) | 0 (0) | 0 (0) | 0 (0) | - | - | 0 (0) | 0 (0) | - | - |
| Vaginal bleeding, No. (%) | 0 (0) | 0 (0) | 0 (0) | - | - | 0 (0) | 0 (0) | - | - |
| Fever, No. (%) | 0 (0) | 0 (0) | 0 (0) | - | - | 0 (0) | 0 (0) | - | - |
| Pessary repositioning, No. (%) |  |  | 1/105 (1) |  |  | 1/103 (1) |  |  |  |

Relative Risk (95% CI) and *p*-values were calculated using the Wald test
