# Supplementary material for: Cervical cerclage versus cervical pessary with or without vaginal progesterone for preterm birth prevention in twin pregnancies and a short cervix: A two-by-two factorial randomised clinical trial
Source: PLoS Med. 2025 Feb 21;22(2):e1004526. doi: 10.1371/journal.pmed.1004526 (PMC11844863; doi:10.1371/journal.pmed.1004526)
Supplement: S6 Table — (DOCX) [file pmed.1004526.s007.docx]

S6 Table: Treatment outcomes in different quartiles of cervical length (intention-to-treat analysis)

|  | **Quartile of cervical length: <25th (13-24 mm)** | | | | | | | |
| --- | --- | --- | --- | --- | --- | --- | --- | --- |
|  | **Cerclage (N=20)** | **Pessary (N=19)** | **Relative Risk  (95% CI)** | **p-values** | **Progesterone (N=19)** | **No Progesterone (N=20)** | **Relative Risk (95% CI)** | **p-values** |
| Preterm birth <34 weeks, No. (%) | 5 (25.0) | 4 (21.1) | 1.19 (0.37-3.77) | 0.787 | 5 (26.3) | 4 (20.0) | 1.32 (0.41-4.18) | 0.663 |
| Composite of poor perinatal outcomes, No. (%) | 6 (30.0) | 7 (36.8) | 0.81 (0.33-1.99) | 0.669 | 5 (26.3) | 8 (40.0) | 0.66 (0.26-1.66) | 0.394 |
| Perinatal death, No. (%) | 1 (5.00) | 2 (10.5) | 0.48 (0.05-4.82) | 0.586 | 2 (10.5) | 1 (5.00) | 2.11 (0.21-21.36) | 0.586 |
|  | **Quartile of cervical length: >=25-50^th^ (25-26 mm)** | | | | | | | |
|  | **Cerclage (N=20)** | **Pessary (N=21)** | **Relative Risk  (95% CI)** | **p-values** | **Progesterone (N=18)** | **No Progesterone (N=23)** | **Relative Risk (95% CI)** | **p-values** |
| Preterm birth <34 weeks, No. (%) | 3 (15.0) | 4 (19.0) | 0.79 (0.2-3.09) | 0.754 | 2 (11.1) | 5 (21.7) | 0.51 (0.11-2.34) | 0.413 |
| Composite of poor perinatal outcomes, No. (%) | 5 (25.0) | 7 (33.3) | 0.75 (0.28-1.98) | 0.582 | 5 (27.8) | 7 (30.4) | 0.91 (0.35-2.4) | 0.866 |
| Perinatal death, No. (%) | 0 (0) | 2 (9.5) | - | - | 0 (0) | 2 (8.7) | - | - |
|  | **Quartile of cervical length: >=50-75^th^ (27 mm)** | | | | | | | |
|  | **Cerclage (N=27)** | **Pessary (N=30)** | **Relative Risk  (95% CI)** | **p-values** | **Progesterone (N=26)** | **No Progesterone (N=31)** | **Relative Risk (95% CI)** | **p-values** |
| Preterm birth <34 weeks, No. (%) | 5 (18.5) | 5 (16.7) | 1.11 (0.36-3.42) | 0.861 | 4 (15.4) | 6 (19.4) | 0.79 (0.25-2.52) | 0.717 |
| Composite of poor perinatal outcomes, No. (%) | 11 (40.7) | 9 (30.0) | 1.36 (0.67-2.77) | 0.414 | 7 (26.9) | 13 (41.9) | 0.64 (0.3-1.37) | 0.254 |
| Perinatal death, No. (%) | 1 (3.70) | 1 (3.3) | 1.11 (0.07-16.91) | 0.947 | 0 (0) | 2 (6.5) | - | - |
|  | **Quartile of cervical length: >=75^th^ (28 mm)** | | | | | | | |
|  | **Cerclage (N=34)** | **Pessary (N=35)** | **Relative Risk  (95% CI)** | **p-values** | **Progesterone (N=40)** | **No Progesterone (N=29)** | **Relative Risk (95% CI)** | **p-values** |
| Preterm birth <34 weeks, No. (%) | 7 (20.6) | 7 (20.0) | 1.03 (0.4-2.62) | 0.953 | 8 (20.0) | 6 (20.7) | 0.97 (0.38-2.49) | 0.940 |
| Composite of poor perinatal outcomes, No. (%) | 12 (35.3) | 10 (28.6) | 1.24 (0.62-2.47) | 0.563 | 15 (37.5) | 7 (24.1) | 1.55 (0.73-3.32) | 0.255 |
| Perinatal death, No, (%) | 0 (0) | 4 (11.4) | - | - | 3 (7.5) | 1 (3.5) | 2.17 (0.24-19.87) | 0.543 |

Relative Risk (95% CI) and *p*-values were calculated using the Wald test
