# Supplementary material for: Cervical cerclage versus cervical pessary with or without vaginal progesterone for preterm birth prevention in twin pregnancies and a short cervix: A two-by-two factorial randomised clinical trial
Source: PLoS Med. 2025 Feb 21;22(2):e1004526. doi: 10.1371/journal.pmed.1004526 (PMC11844863; doi:10.1371/journal.pmed.1004526)
Supplement: S7 Table — (DOCX) [file pmed.1004526.s008.docx]

S7 Table: Best and worst case scenarios of PTB <34 weeks of all randomised participants

|  | Cervical cerclage (N=109) | Pessary (N=110) | Relative Risk  (95% CI) | p-values | Progesterone  (N=111) | No Progesterone (N=108) | Relative Risk  (95% CI) | p-values |
| --- | --- | --- | --- | --- | --- | --- | --- | --- |
| Best case for PTB<34 wks, No. (%) | 20 (18.4) | 20 (18.1) | 1.01  (0.58-1.77) | 0.975 | 19 (17.1) | 21 (19.4) | 0.88  (0.50-1.54) | 0.661 |
| Worst case for PTB<34 wks, No. (%) | 28 (25.7) | 25 (22.7) | 1.13  (0.71-1.81) | 0.614 | 27 (24.3) | 26 (24.1) | 1.01  (0.63-1.61) | 0.966 |

A best-case scenario assuming all women lost to follow-up, withdraw, and had no intervention were did not have preterm birth <34 weeks.

A worst-case scenario assuming all women lost to follow-up, withdraw, and had no intervention had preterm birth <34 weeks.

Relative Risk (95% CI) and *p*-values were calculated using the Wald test
