# Supplementary material for: Cervical cerclage versus cervical pessary with or without vaginal progesterone for preterm birth prevention in twin pregnancies and a short cervix: A two-by-two factorial randomised clinical trial
Source: PLoS Med. 2025 Feb 21;22(2):e1004526. doi: 10.1371/journal.pmed.1004526 (PMC11844863; doi:10.1371/journal.pmed.1004526)
Supplement: S8 Table — (DOCX) [file pmed.1004526.s009.docx]

S8 Table: Outcomes on maternal level (per-protocol analysis)

|  |  | Pessary vs Cervical cerclage | | | | Progesterone vs No Progesterone | | | |
| --- | --- | --- | --- | --- | --- | --- | --- | --- | --- |
|  | All (N=203) | Cerclage (N=102) | Pessary (N=101) | Relative Risk  (95% CI) | p-values | Progesterone (N=106) | No Progesterone (N=97) | Relative Risk (95% CI) | p-values |
| Primary outcome | | | | | | | | | |
| Preterm birth <34 wk, No. (%) | 40 (19.7) | 20 (19.6) | 20 (19.8) | 0.99 (0.57-1.73) | 0.973 | 19 (17.9) | 21 (21.6) | 0.83 (0.47-1.44) | 0.511 |
| Secondary outcomes | | | | | | | | | |
| Miscarriage, No. (%) | 1 (0.5) | 0 (0.0) | 1 (1.0) | - | - | 1 (0.9) | 0 (0.0) | - | - |
| Stillbirth ≥28 wk, No. (%) | 1 (0.5) | 0 (0.0) | 1 (1.0) | - | - | 0 (0.0) | 1 (1.0) | - | - |
| Stillbirth <28 wk, No. (%) ^a^ | 8 (3.9) | 1 (1.0) | 7 (6.9) | 0.14 (0-0.66) | 0.035 | 5 (4.7) | 3 (3.1) | 1.16 (0.28-4.71) | 0.581 |
| Stillbirth <34 wk, No. (%) ^a^ | 9 (4.4) | 1 (1.0) | 8 (7.9) | 0.12 (0-0.59) | 0.018 | 5 (4.7) | 4 (4.1) | 1.14 (0.23-6.41) | 0.851 |
| Neonatal death <24 wk, No. (%) | 2 (1.0) | 0 (0.0) | 2 (1.0) | - | - | 2 (1.0) | 0 (0.0) | - | - |
| Perinatal death, No. (%) ^b^ | 11 (5.4) | 2 (2.0) | 9 (8.9) | 0.22 (0-0.74) | 0.033 | 5 (4.7) | 6 (6.2) | 0.76 (0.18-2.75) | 0.669 |
| Preterm birth <24 wk, No. (%) | 3 (1.5) | 0 (0.0) | 3 (3.0) | - | - | 3 (2.8) | 0 (0.0) | - | - |
| Preterm birth <28 wk, No. (%) | 10 (4.9) | 1 (1.0) | 9 (8.9) | 0.11 (0-0.50) | 0.010 | 6 (5.7) | 4 (4.1) | 1.37 (0.37-7.32) | 0.635 |
| Preterm birth <32 wk, No. (%) | 21 (10.3) | 9 (8.8) | 12 (11.9) | 0.74 (0.33-1.69) | 0.486 | 14 (13.2) | 7 (7.2) | 1.83 (0.77-4.34) | 0.171 |
| Preterm birth <37 wk, No. (%) | 124 (61.1) | 66 (64.7) | 58 (57.4) | 1.13 (0.9-1.41) | 0.292 | 67 (63.2) | 57 (58.8) | 1.08 (0.86-1.34) | 0.521 |
| Spontaneous preterm birth <28 wk, No. (%) | 9 (4.4) | 1 (1.0) | 8 (7.9) | 0.12 (0-0.59) | 0.018 | 6 (5.7) | 3 (3.1) | 1.39 (0.36-5.39) | 0.403 |
| Spontaneous preterm birth <34 wk, No. (%) | 38 (18.7) | 19 (18.6) | 19 (18.8) | 0.99 (0.56-1.76) | 0.974 | 19 (17.9) | 19 (19.6) | 0.92 (0.52-1.62) | 0.764 |
| Spontaneous preterm birth <37 wk, No. (%) | 92 (45.3) | 52 (51.0) | 40 (39.6) | 1.29 (0.95-1.75) | 0.107 | 53 (50.0) | 39 (40.2) | 1.24 (0.91-1.69) | 0.165 |
| Iatrogenic preterm birth <28 wk, No. (%) | 1 (0.5) | 0 (0.0) | 1 (1.0) | - | - | 0 (0.0) | 1 (1.0) | - | - |
| Iatrogenic preterm birth <34 wk, No. (%) | 2 (1.0) | 1 (1.0) | 1 (1.0) | 0.50 (0.03-7.89) | 0.995 | 0 (0.0) | 2 (2.1) | - | - |
| Iatrogenic preterm birth <37 wk, No. (%) | 32 (15.8) | 14 (13.7) | 18 (17.8) | 0.77 (0.41-1.46) | 0.432 | 14 (13.2) | 18 (18.6) | 0.71 (0.37-1.35) | 0.305 |
| Onset of labor, No. (%) ^c^ | 98 (48.5) | 52 (51.0) | 46 (46.0) | 1.11 (0.83-1.47) | 0.483 | 59 (56.2) | 39 (40.2) | 1.4 (1.04-1.88) | 0.024 |
| Mode of delivery, No. (%) ^c^ |  |  |  |  |  |  |  |  |  |
| C-section | 192 (94.6) | 100 (98.0) | 92 (91.1) | 1.08 (1.01-1.15) | 0.033 | 101 (95.3) | 91 (93.8) | 1.02 (0.95-1.09) | 0.659 |
| Elective | 105 (54.7) | 52 (52.0) | 53 (57.6) | - | 0.398^d^ | 48 (47.5) | 57 (62.6) | - | 0.052^d^ |
| Nonprogressive labor | 86 (44.8) | 48 (48.0) | 38 (41.3) | - | - | 53 (52.5) | 33 (36.3) | - | - |
| Suspected fetal distress | 1 (0.5) | 0 (0.0) | 1 (1.1) | - | - | 0 (0.0) | 1 (1.1) | - | - |
| Live birth, n(%) | 201 (99.0) | 102 (100.0) | 99 (98.0) | 1.02 (0.99-1.05) | 0.246 | 104 (98.1) | 97 (100.0) | 0.98 (0.96-1.01) | 0.271 |
| Gestational age at delivery, mean (SD), wk ^c^ | 35.3 (3.2) | 35.6 (2.4) | 35.0 (3.9) | - | 0.191^e^ | 35.1 (3.6) | 35.5 (2.7) | - | 0.341^e^ |
| Time from randomization to delivery, median (Q1-Q3),  ^c^ | 127 (111-138.8) | 126.5 (111-137.8) | 127.5 (110.8-140) | - | 0.872^f^ | 127 (111-137) | 128 (111-141) | - | 0.518^f^ |

^a^ post-hoc analysis; ^b^ defined as stillbirth ≥20 weeks and neonatal death ≥20 weeks;  ^c^ 1 case miscarriage, *p*-values according to a dichotomous outcome were calculated using the Wald test. ^d^*p*-values were calculated using the Chi-squared test, ^e^*p*-values were calculated using the T-Test, ^f^*p*-values were calculated using the Mann–Whitney U test
