# Supplementary material for: Cervical cerclage versus cervical pessary with or without vaginal progesterone for preterm birth prevention in twin pregnancies and a short cervix: A two-by-two factorial randomised clinical trial
Source: PLoS Med. 2025 Feb 21;22(2):e1004526. doi: 10.1371/journal.pmed.1004526 (PMC11844863; doi:10.1371/journal.pmed.1004526)
Supplement: S9 Table — (DOCX) [file pmed.1004526.s010.docx]

S9 Table: Outcomes on neonatal level (per-protocol analysis)

|  |  | Cerclage versus Pessary | | | | Progesterone vs No Progesterone | | | |  |
| --- | --- | --- | --- | --- | --- | --- | --- | --- | --- | --- |
|  | All (N=404) | Cerclage (N=204) | Pessary (N=200) | Relative Risk  (95% CI) | p-values | Progesterone (N=210) | No Progesterone (N=194) | Relative Risk (95% CI) | p-values | |
| Stillbirth ≥28 weeks, No. (%) | 1 (0.2) | 0 (0.0) | 1 (0.5) | - | - | 0 (0.0) | 1 (0.5) | - | - | |
| Stillbirth <28 weeks, No. (%) ^a^ | 9 (2.2) | 1 (0.5) | 8 (4.0) | 0.12 (0.02-0.72) | 0.05 | 6 (2.9) | 3 (1.5) | 1.85 (0.55-6.16) | 0.402 | |
| Stillbirth <34 weeks, No. (%) ^a^ | 10 (2.5) | 1 (0.5) | 9 (4.5) | 0.11 (0.02-0.62) | 0.036 | 6 (2.9) | 4 (2.1) | 1.39 (0.46-4.19) | 0.627 | |
| Neonatal death <24 weeks, No. (%) | 2 (0.5) | 0 (0.0) | 2 (1.0) | - | - | 2 (1.0) | 0 (0.0) | - | - | |
| Perinatal death, No (%) ^b^ | 14 (3.5) | 2 (1.0) | 12 (6.0) | 0.16 (0.05-0.59) | 0.020 | 8 (3.8) | 6 (3.1) | 1.23 (0.46-3.33) | 0.730 | |
| Birth weight, mean (SD), g ^c^ | 2248 (581) | 2288 (498) | 2207 (654) | - | 0.164^f^ | 2232 (626) | 2265 (530) | - | 0.567^f^ | |
| Birth weight <1500 g, No. (%) ^c^ | 39 (9.7) | 13 (6.4) | 26 (13.1) | 0.49 (0.24-1.02) | 0.110 | 24 (11.4) | 15 (7.8) | 1.48 (0.74-2.94) | 0.350 | |
| Birth weight <2500 g, No. (%) ^c^ | 238 (59.1) | 126 (61.8) | 112 (56.3) | 1.10 (0.94-1.30) | 0.329 | 128 (61.0) | 110 (57.0) | 1.07 (0.91-1.27) | 0.474 | |
| 5-min Apgar score, median (Q1-Q3) ^d^ | 9.0 (8.0-9.0) | 9.0 (8.0-9.0) | 9.0 (8.0-9.0) | - | 0.262^g^ | 9.0 (8.0-9.0) | 9.0 (8.0-9.0) | - | 0.300^g^ | |
| Apgar score <7, No. (%) ^d^ | 8 (3.2) | 1 (0.8) | 7 (5.8) | 0.14 (0.02-0.88) | 0.078 | 4 (3.2) | 4 (3.2) | 0.92 (0.20-4.23) | 0.932 | |
| Congenital anomalies after randomization, No. (%) | 8 (2.0) | 7 (3.4) | 1 (0.5) | 6.86 (1.13-41.74) | 0.079 | 2 (1.0) | 6 (3.1) | 0.31 (0.07-1.29) | 0.176 | |
| Admission to NICU, No. (%) | 140 (34.7) | 74 (36.3) | 66 (33.0) | 1.10 (0.81-1.49) | 0.608 | 68 (32.4) | 72 (37.1) | 0.87 (0.64-1.18) | 0.460 | |
| Length of NICU admission, median (Q1-Q3), d ^e^ | 3.0 (2.0-10.0) | 3.0 (2.0-12.8) | 2.0 (2.0-9.0) | - | 0.127^g^ | 3.0 (2.0-9.0) | 3.0 (2.0-12.2) | - | 0.833^g^ | |
| Respiratory distress syndrome, No. (%) | 89 (22.0) | 52 (25.5) | 37 (18.5) | 1.38 (0.92-2.06) | 0.192 | 47 (22.4) | 42 (21.6) | 1.03 (0.69-1.54) | 0.892 | |
| Intraventricular haemorrhage, No. (%) | 1 (0.2) | 0 (0.0) | 1 (0.5) | - | - | 1 (0.5) | 0 (0.0) | - | - | |
| Necrotizing enterocolitis, No. (%) | 11 (2.7) | 8 (3.9) | 3 (1.5) | 2.61 (0.65-10.59) | 0.258 | 8 (3.8) | 3 (1.5) | 2.46 (0.61-9.98) | 0.289 | |
| Proven sepsis, No. (%) | 38 (9.4) | 22 (10.8) | 16 (8.0) | 1.35 (0.72-2.52) | 0.432 | 14 (6.7) | 24 (12.4) | 0.54 (0.28-1.04) | 0.120 | |
| Composite of poor perinatal outcomes, No. (%) | 104 (25.7) | 59 (28.9) | 45 (22.5) | 1.29 (0.90-1.83) | 0.245 | 52 (24.8) | 52 (26.8) | 0.92 (0.65-1.32) | 0.714 | |

NICU: Neonatal intensive care unit; ^a^ post-hoc analysis; ^b^ defined as any stillbirth ≥20 weeks and neonatal death ≥20 weeks;  ^c^ 1 case with missing data excluded; ^d^ 152 cases with missing data (out of 404 babies) excluded; ^e^ 6 cases with missing data (out of 140 NICU admission) excluded. . *p*-values according to a dichotomous outcome were calculated using GEE model. ^f^p-values were calculated using the T-Test, ^g^p-values were calculated using the Mann–Whitney U test
