# Supplementary material for: Cervical cerclage versus cervical pessary with or without vaginal progesterone for preterm birth prevention in twin pregnancies and a short cervix: A two-by-two factorial randomised clinical trial
Source: PLoS Med. 2025 Feb 21;22(2):e1004526. doi: 10.1371/journal.pmed.1004526 (PMC11844863; doi:10.1371/journal.pmed.1004526)
Supplement: S10 Table — (DOCX) [file pmed.1004526.s011.docx]

S10 Table: Treatment outcomes in different quartiles of cervical length (per-protocol analysis)

|  | **Quartile of cervical length: <25^th^ (13-24 mm)** | | | | | | | |
| --- | --- | --- | --- | --- | --- | --- | --- | --- |
|  | **Cerclage (N=21)** | **Pessary (N=17)** | **Relative Risk (95% CI)** | **p-values** | **Progesterone (N=17)** | **No Progesterone (N=21)** | **Relative Risk (95% CI)** | **p-values** |
| Preterm birth <34 weeks, No. (%) | 5 (23.8) | 4 (23.5) | 1.01 (0.32-3.19) | 0.989 | 5 (29.4) | 4 (19.0) | 1.54 (0.49-4.87) | 0.485 |
| Composite of poor perinatal outcomes, No. (%) | 6 (28.6) | 7 (41.2) | 0.69 (0.29-1.68) | 0.442 | 5 (29.4) | 8 (38.1) | 0.77 (0.31-1.93) | 0.599 |
| Perinatal death, No. (%) | 1 (4.76) | 2 (11.8) | 0.4 (0.04-4.09) | 0.500 | 2 (11.8) | 1 (4.76) | 2.47 (0.24-24.98) | 0.500 |
|  | **Quartile of cervical length: >=25-50^th^ (25-26 mm)** | | | | | | | |
|  | **Cerclage (N=20)** | **Pessary (N=19)** | **Relative Risk (95% CI)** | **p-values** | **Progesterone (N=19)** | **No Progesterone (N=20)** | **Relative Risk (95% CI)** | **p-values** |
| Preterm birth <34 weeks, No. (%) | 3 (15.0) | 4 (21.1) | 0.71 (0.18-2.77) | 0.652 | 2 (10.5) | 5 (25.0) | 0.42 (0.09-1.92) | 0.278 |
| Composite of poor perinatal outcomes, No. (%) | 5 (25.0) | 6 (31.6) | 0.79 (0.29-2.17) | 0.668 | 5 (26.3) | 6 (30.0) | 0.88 (0.32-2.4) | 0.812 |
| Perinatal death, No. (%) | 0 (0) | 2 (10.5) | - | - | 0 (0) | 2 (10.0) | - | - |
|  | **Quartile of cervical length: >=50-75^th^ (27 mm)** | | | | | | | |
|  | **Cerclage (N=27)** | **Pessary (N=30)** | **Relative Risk (95% CI)** | **p-values** | **Progesterone (N=28)** | **No Progesterone (N=29)** | **Relative Risk (95% CI)** | **p-values** |
| Preterm birth <34 weeks, No. (%) | 5 (18.5) | 5 (16.7) | 1.11 (0.36-3.42) | 0.861 | 4 (14.3) | 6 (20.7) | 0.69 (0.22-2.19) | 0.551 |
| Composite of poor perinatal outcomes, No. (%) | 11 (40.7) | 9 (30.0) | 1.36 (0.67-2.77) | 0.414 | 7 (25.0) | 13 (44.8) | 0.56 (0.26-1.19) | 0.130 |
| Perinatal death, No. (%) | 1 (3.70) | 1 (3.33) | 1.11 (0.07-16.91) | 0.947 | 0 (0) | 2 (6.90) | - | - |
|  | **Quartile of cervical length: >=75^th^ (28 mm)** | | | | | | | |
|  | **Cerclage (N=34)** | **Pessary (N=35)** | **Relative Risk (95% CI)** | **p-values** | **Progesterone (N=42)** | **No Progesterone (N=27)** | **Relative Risk (95% CI)** | **p-values** |
| Preterm birth <34 weeks, No. (%) | 7 (20.6) | 7 (20.0) | 1.03 (0.4-2.62) | 0.953 | 8 (19.0) | 6 (22.2) | 0.86 (0.33-2.2) | 0.753 |
| Composite of poor perinatal outcomes, No. (%) | 12 (35.3) | 10 (28.6) | 1.24 (0.62-2.47) | 0.563 | 15 (35.7) | 7 (25.9) | 1.38 (0.65-2.93) | 0.412 |
| Perinatal death, No. (%) | 0 (0) | 4 (11.4) | - | - | 3 (7.14) | 1 (3.70) | 1.93 (0.21-17.6) | 0.618 |

Relative Risk (95% CI) and *p*-values were calculated using the Wald test
