# Supplementary material for: Cervical cerclage versus cervical pessary with or without vaginal progesterone for preterm birth prevention in twin pregnancies and a short cervix: A two-by-two factorial randomised clinical trial
Source: PLoS Med. 2025 Feb 21;22(2):e1004526. doi: 10.1371/journal.pmed.1004526 (PMC11844863; doi:10.1371/journal.pmed.1004526)
Supplement: S11 Table — (DOCX) [file pmed.1004526.s012.docx]

S11 Table: PTB <28 weeks’ gestation in different quartiles of cervical length

| **Cervical length (mm)** | **Cerclage** | **Pessary** | **Progesterone** | **No progesterone** |
| --- | --- | --- | --- | --- |
| 13-24 | 1/20 | 1/19 | 2/19 | 0/20 |
| 25-26 | 0/20 | 2/21 | 0/18 | 2/23 |
| 27 | 0/27 | 2/30 | 1/26 | 1/31 |
| 28 | 0/34 | 4/35 | 3/40 | 1/29 |
