# Supplementary material for: Cervical cerclage versus cervical pessary with or without vaginal progesterone for preterm birth prevention in twin pregnancies and a short cervix: A two-by-two factorial randomised clinical trial
Source: PLoS Med. 2025 Feb 21;22(2):e1004526. doi: 10.1371/journal.pmed.1004526 (PMC11844863; doi:10.1371/journal.pmed.1004526)
Supplement: S12 Table — (DOCX) [file pmed.1004526.s013.docx]

S12 Table. Secondary outcomes’ definition

| **Obstetrics outcomes** | |
| --- | --- |
| Fetal death before 24 weeks’ gestation | Fetal death before 24 weeks’ gestation |
| Stillbirth | Fetus born with no signs of life at or after 28 weeks' gestation |
| Gestational age at delivery | For women who conceived after ART, gestational age will be determined by the date of embryo transfer or intrauterine insemination. For those who conceived naturally, gestational age will be determined from the menstrual history and confirmed by the fetal crown-rump length of the larger twin at the first-trimester ultrasound examination. |
| Time from randomization to delivery | Time interval between date of randomization and date of delivery, weeks |
| Preterm birth <24 weeks | Any birth from randomization to 23 6/7 weeks |
| Preterm birth <28 weeks | Any birth from randomization to 27 6/7 weeks |
| Preterm birth <32 weeks | Any birth from randomization to 31 6/7 weeks |
| Preterm birth <37 weeks | Any birth from randomization to 36 6/7 weeks |
| Spontaneous preterm birth <28 weeks | Birth spontaneously from randomization to 27 6/7 weeks, including preterm spontaneous rupture of membranes, preterm premature rupture of membranes (PPROM) |
| Spontaneous preterm birth <34 weeks | Birth spontaneously from randomization to 33 6/7 weeks, including preterm spontaneous rupture of membranes, preterm premature rupture of membranes (PPROM) |
| Spontaneous preterm birth <37 weeks | Birth spontaneously from randomization to 36 6/7 weeks, including preterm spontaneous rupture of membranes, preterm premature rupture of membranes (PPROM) |
| Iatrogenic preterm birth <28 weeks | Birth non-spontaneously from randomization to 27 6/7 weeks |
| Iatrogenic preterm birth <34 weeks | Birth non-spontaneously from randomization to 33 6/7 weeks |
| Iatrogenic preterm birth <37 weeks | Birth non-spontaneously from randomization to 36 6/7 weeks |
| Onset of labor | Spontaneous or labor induction or elective C-section |
| Labor induction | Whether the participant underwent labor induction or not |
| Mode of delivery | Vaginal delivery or C-section (elective, suspected fetal distress or non-progressive labor) |
| Livebirth | The birth of at least one newborn, regardless of gestational age, that exhibits any sign of life such as respiration, heartbeat, umbilical pulsation or movement of voluntary muscles |
| Use of tocolytic drugs | Use of any tocolytic drug to treat preterm labor, from 24 0/7 to 33 6/7 weeks' gestation |
| Use of antenatal corticosteroids | Use of antenatal corticosteroids to prevent respiratory distressed syndrome, from 24 0/7 to 33 6/7 weeks' gestation |
| Use of magnesium sulfate for neuroprotection | Use of magnesium sulfate for neuroprotection, from 28 0/7 to 31 6/7 weeks' gestation |
| Length of maternal admission for preterm labor | Number of admission days for treatment of preterm labor, from 24 0/7 to 37 0/7 weeks |
| Preterm prelabor rupture of membranes | Prelabor rupture of membranes, from randomization to less than 37 0/7 weeks |
| Chorioamnionitis | Intraamniotic infection, from randomization to delivery |
| Maternal side effects | Including vaginal discharge, fever, vaginal bleeding, vaginal infection (confirmed by vaginal discharge culture), vaginal pain, pessary repositioning and necrosis or rupture of the cervix, from date of randomization until delivery |
| Maternal morbidity | Including thromboembolic complications, urinary tract infection treated with antibiotics, pneumonia, endometritis, hypertensive disorder, eclampsia, hemolysis, elevated liver enzymes, low platelet count syndrome |
| Maternal mortality | Death of the mother, from randomization to delivery |
| **Neonatal outcomes** | |
| Birthweight | Weight of baby born at birth, gram |
| Birthweight <1500 g | Weight of baby born at birth <1500 g |
| Birthweight <2500 g | Weight of baby born at birth <2500 g |
| Congenital anomalies after randomization | Any congenital anomalies detected in baby born |
| 5-min Apgar score | Apgar score at 5 minutes after birth |
| 5-min Apgar score <7 | Apgar score at 5 minutes after birth <7 |
| Perinatal death | Stillbirth or neonatal death of a baby of 20 or more completed weeks of gestation |
| Admission to neonatal intensive care unit (NICU) | Admission to neonatal intensive care unit of baby, within 7 days after birth |
| Length of NICU admission | Number of admission days to NICU, up to 28 days after birth |
| Intraventricular hemorrhage II B or worse | Repeated neonatal cranial ultrasound by the neonatologist according to the guidelines on neuro-imaging described by de Vries et al, up to 28 days after birth |
| Respiratory distress syndrome | The presence of tachypnoea >60/minute, sternal recession and expiratory grunting, need for supplemental oxygen, and a radiological picture of diffuse reticulogranular shadowing with an air bronchogram, up to 28 days after birth |
| Necrotizing enterocolitis | Diagnosed according to Bell, up to 28 days after birth |
| Proven sepsis | The combination of clinical signs and positive blood cultures, up to 28 days after birth |
| Composite of poor perinatal outcomes | Fetal or neonatal death, intraventricular hemorrhage, respiratory distress syndrome, necrotizing enterocolitis or neonatal sepsis, up to 28 days after birth |
| Death before discharge | Death of newborn before discharge from nursery, up to 28 days after birth |
