# Supplementary material for: Cervical cerclage versus cervical pessary with or without vaginal progesterone for preterm birth prevention in twin pregnancies and a short cervix: A two-by-two factorial randomised clinical trial
Source: PLoS Med. 2025 Feb 21;22(2):e1004526. doi: 10.1371/journal.pmed.1004526 (PMC11844863; doi:10.1371/journal.pmed.1004526)
Supplement: S1 Protocol — (DOCX) [file pmed.1004526.s014.docx]

**PROTOCOL**

**The effectiveness of**

**cervical pessary compared to cervical cerclage**

**with or without vaginal progesterone for**

**the prevention of preterm birth**

**in women with a twin pregnancy and a short cervix:**

**A two-by-two factorial randomised clinical trial**

**(04 Jan 2022)**

| Protocol ID |  |
| --- | --- |
| Short title | **P**essary versus **Ce**rclage with or without **P**rogesterone for prevention of preterm birth in twins (PCP-Twins) |
| Version | 4.0 |
| Date | 04 Jan 2022 |
| Coordinating investigator/project leader | Dr He Thanh Nha Yen |
| Principal investigator(s) | Dr Pham Nguyen Hoa Ha  My Duc Hospital, HCMC, Viet Nam  Mr Nguyen Cao Tri  My Duc Hospital, HCMC, Viet Nam  Dr Bui Quang Trung  My Duc Hospital, HCMC, Viet Nam  Dr Vuong Tu Nhu  My Duc Hospital, HCMC, Viet Nam  Mrs Nguyen Thi Ngoc Diem  My Duc Hospital, Viet Nam  Dr Le Van Thanh  My Duc Hospital, Viet Nam  Dr. Wentao Li  Monash University, Australia  A/Prof Le Hong Cam  My Duc Hospital. HCMC, Viet Nam  Dr Ho Manh Tuong  My Duc Hospital, HCMC, Viet Nam  Prof Dr. B.W.J. Mol  Monash University, Australia  Dr Dang Quang Vinh  Monash University, Australia  Dr Vuong Thi Ngoc Lan  University of Medicine and Pharmacy at Ho Chi Minh City |
| Sponsor | My Duc Hospital |

SUMMARY

**Rationale:** Preterm birth (PTB) is one of the dominant causes of neonatal mortality and morbidity in women with a twin pregnancy. However, at present there is no clearly effective strategy to prevent PTB in this situation. In our previous AP study, we found cervical pessary to be superior over vaginal progesterone in the reduction of PTB <34 weeks and in the reduction of a composite of poor perinatal outcomes in women with a cervical length (CL) ≤28 mm. Therefore, we here compare the effectiveness of cervical pessary to cervical cerclage with or without vaginal progesterone in women with a twin pregnancy and a CL ≤28 mm.

**Objectives:**

To compare the effectiveness of cervical pessary and cervical cerclage in preventing PTB in women with an asymptomatic twin pregnancy and a midtrimester cervix ≤28 mm.

To assess the effectiveness of progesterone in preventing PTB in women with an asymptomatic twin pregnancy and a midtrimester cervix ≤28 mm.

**Study design:** Open label, multi-center, two-by-two factorial, randomised clinical trial.

**Setting:** Ob/Gyn department, My Duc Hospital, My Duc Phu Nhuan Hospital.

**Study population:** Pregnant women having a twin pregnancy from 16 ^0/7^ to 22 ^0/7^ weeks and a CL ≤28 mm.

**Interventions:** Randomisation will occur after eligibility assessment and informed consent. Patients will be randomised to either cerclage, pessary, cerclage plus progesterone or pessary plus progesterone in a 1:1:1:1 ratio with a variable block size of 4 or 8. Based on the randomisation results, an Arabin pessary or cerclage or cerclage plus progesterone or pessary plus progesterone will be placed within a week after randomisation. Interventions will be continued until 37^0/7^ weeks of gestation or delivery.

**Primary outcome measure:** PTB before 34 weeks of gestation for any indication.

**Power calculation:** The prevalence of PTB <34 weeks of gestation in women with a twin pregnancy and a cervix ≤28 mm in our previous AP study at My Duc hospital was 24.2% in the pessary group. In order to show or refute that cervical cerclage decreases the PTB rate by 50% (from 24.2% to 12.1%), we need to randomise 320 women (alpha level 0.05, power 80%). The statistical power to study the effect of progesterone by randomizing 320 women is 80% (alpha level 0.05, PTB rate in progesterone group 54.5%, absolute rate difference 15.5%). Considering a 5% loss to follow up and protocol violation we plan to recruit 340 participants (85 per arm).

**Nature and extent of the burden and risks associated with participation, benefit and group relatedness:** As we evaluate strategies that are already applied in current practice for singleton pregnancies and we used cervical pessary in our AP study, no additional risks or burdens are expected from the study.

1. INTRODUCTION AND RATIONALE

Preterm birth (PTB) is the most common cause of neonatal morbidity and mortality worldwide (*Office for National Statistics*, 2012; Saigal & Doyle, 2008) and the second-leading cause of death in children under 5 years (Blencowe et al., 2012; Liu et al., 2012). In Viet Nam, a report from 2012 showed that out of 1,466,600 live births, there were 138,300 (9.4%) babies born preterm (Blencowe et al., 2012). That report also showed that Viet Nam was ranked 21^st^ in the world for number of PTB. At present, there are three main risk indicators that are used to identify women at risk for spontaneous preterm birth, i.e. women with a previous PTB, women with a multiple pregnancy and women with a midtrimester short cervix as identified on transvaginal sonography. Women with a twin pregnancy have a 50% risk of delivering before 37 weeks. In addition, short cervical length (CL) in the second trimester of pregnancy is well known to be an independent risk factor for preterm birth(Iams et al., 1996). Therefore, women with a multiple pregnancy and a short cervix are under extremely high risk for PTB.

Cervical cerclage (Abdel-Aleem et al., 2013; Saccone et al., 2015), cervical pessaries (Goya et al., 2016; Houlihan et al., 2016; Liem et al., 2013; Nicolaides et al., 2016; Saccone et al., 2017) and vaginal progesterone (Jarde et al., 2017; Romero et al., 2017; Schuit et al., 2015) (are all promising in preventing PTB but more comparative effectiveness data are needed. A meta-analysis on three trials with 49 women with a twin gestation with a short cervical length could not demonstrate a benefit of cerclage in preventing PTB in women with a twin pregnancy and a cervical length <25 mm (Saccone et al., 2015). Moreover, cerclage group had higher rates of very low birthweight and of respiratory distress syndrome than control group. On the other hand, a recent retrospective cohort study showed that in women with a twin pregnancy and a CL ≤15 mm, cerclage could reduce the PTB <35 weeks rate significantly from 79.5% to 50% aOR, 0.51; 95% CI, 0.31-0.83 as well as the rate of admission to NICU, from 82.9% to 65.5% aOR, 0.42; 95% CI, 0.24-0.81 (Roman et al., 2015). These positive findings were also confirmed in other two recent studies (Adams et al., 2018; Houlihan et al., 2016). However, the sample size in the above mentioned studies was relatively limited.

Among types of cervical pessaries, the Arabin is the most common used. This pessary has shown encouraging results in women with twin pregnancy. A large open-label RCT (Liem et al., 2013) was conducted at 40 Dutch centers from 2009 to 2012 on 813 women with a twin pregnancy. While overall there was no effect, in women with a short cervix (<25% percentile, equal to 38 mm), pessary reduced PTB <28 weeks (p = 0.0158) and <32 weeks (p = 0.0476), median of time to delivery (p = 0.0437), thus improving neonatal outcomes (p = 0.0106). These findings were confirmed by Goya et al.(Goya et al., 2016). However, data from a subgroup analysis in twin pregnant women with a short cervix showed no effect with cervical pessary (Nicolaides et al., 2016).

In view of the large problem of PTB in women with a twin pregnancy and in view of the promising effect of both vaginal progesterone and cervical pessary, we recently performed the AP study in our center. In this study, we compared the effectiveness of cervical pessary and 400mg progesterone daily in women with a twin pregnancy and a cervical length <38mm. While overall no significant effect of cervical pessary was found, in women with a cervical length ≤28 mm, in women with a CL <25th percentile (≤28 mm), the PTB rate <34 reduced from 54.5% in the progesterone group to 24.2% in the pessary group and (RR 0.44, 95% CI 0.22 to 0.91, p=0.04). In women with a CL between 28mm and 38mm, there were no significant differences in preterm birth rate (Dang et al., 2018).

As we think that these data showed that below a CL of 28 mm, cervical pessary is superior to progesterone, this question that arises now is how cervical pessary compares to cervical cerclage. We therefore propose the Pessary versus Cerclage with or without Progesterone study, in which we will compare the effectiveness of cervical pessary and cervical cerclage with or without vaginal progesterone to prevent PTB in women with a twin pregnancy and a CL ≤28mm (PCP-Twins).

2. OBJECTIVES

Primary objective: To compare the effectiveness of cervical pessary to cervical cerclage for prevention of PTB in women with a twin pregnancy and a cervix ≤28 mm.

Secondary objective: To determine the effectiveness of vaginal progesterone for the prevention of PTB in women with a twin pregnancy and a cervix ≤28 mm.

3. STUDY DESIGN

A multi-center, two-by-two factorial, randomised clinical trial. The analysis would follow the intention-to-treat principle.

Flowchart of the study:

Women eligible for trial

(Twin pregnancies, 16 0/7 to 22 0/7 weeks, Cervical length ≤28 mm)

Informed consent

Assessment of exclusion criteria

Placenta previa

Fetal congenital anomalies or death or poor fetal condition

Premature rupture of membranes

Other adverse obstetric conditions

Cervical cerclage

Randomisation

Cervical cerclage + vaginal progesterone

Cervical pessary + vaginal progesterone

Cervical pessary

Treat according to local protocol

**Registration of outcomes**

Obstetric outcomes and fetal outcomes

Yes

No

4. STUDY POPULATION

**Inclusion and exclusion criteria**

**Inclusion criteria**

- Women with a twin pregnancy (mono- and di-chorionic)
- 16 ^0/7^ to 22 ^0/7^ weeks of gestation
- Maternal age ≥18 years
- Cervical length ≤28 mm
- Informed consent
- Not participating in another PTB study at the same time

**Exclusion criteria**

- Uterine anomalies
- Cervical dilation with visible amniotic membranes or amniotic membranes prolapsed into the vagina
- Twin-to-twin transfusion syndrome
- Stillbirth or major congenital abnormalities in any of the fetus
- Severe vaginal discharge
- Acute vaginitis or cervicitis
- Vaginal bleeding
- Placenta preavia
- Vasa preavia
- Premature rupture of membranes
- Premature labor with/without ruptured membrane
- Suspicion of chorioamnionitis
- Cerclage or pessary in place or unable to undergo cervical cerclage or pessary with or without vaginal progesterone

5. INFORMED CONSENT, RANDOMISATION, AND WITHDRAW

### 5.1 Subject informed consent

A review of patient information will be done prior to enrollment to determine preliminary eligibility according to patient inclusion and exclusion criteria. Eligible participants will be screened by midwives or gynaecologists, then will be provided a full Patient Information Sheet, Consent Form and invited to a full discussion with investigators about the study. When a patient signs an informed consent, she is considered to be enrolled into the study.

## 5.2 Randomisation

After written informed consent, women will be randomly assigned in a 1:1:1:1 ratio to receive a cerclage, pessary, cerclage plus progesterone or pessary plus progesterone. Randomisation will be centrally controlled by administrative staffs in the trial centre, who are not involved in any treatment procedure. Assignment to treatment allocation will be done via a web portal hosted by HOPE Research Center, Vietnam. The randomisation schedule will be computer-generated at HOPE Research Center, with a permuted random block size of 4 or 8. Blinding will not be possible due to the nature of interventions. However, neonatologists assessing the children will be unaware of treatment allocation. Apart from randomisation, patients will be followed up and treated according to local protocol.

## 5.3 Withdrawal of individual subjects

Patients can leave the study at any time for any reason if they wish to do so without any consequences for their treatment. The investigator can decide to withdraw a subject from the study for urgent medical reasons. After randomisation, if a patient wishes to change her assigned protocol, she will be considered as a cross-over subject. In case a patient wishes to have two or more interventions, after randomisation, she will be considered as a mixing subject. All subjects will be remained in the study for analysis based on intention to treat principle.

6. Study procedures

## 6.1 Pre-randomisation examination and assessment

All women with a twin pregnancy will undergo cervical length measurement and digital examination at screening. CL will be measured transvaginally by two ultrasonographers certificated by the Fetal Medicine Foundation at each hospital. Prior to CL measurement, women will be given a short brochure outlining risk factors and available PTB prevention methods. Only women with a CL ≤28 mm will be eligible for the study. Eligible women will further undergo a speculum examination to assess the feasibility of treatment with either cerclage or cervical pessary with or without progesterone and to exclude premature rupture of the membranes (PROM), acute vaginitis and cervicitis. Only women in whom the clinician assesses treatments as feasible will be randomised.

For women conceived after Assisted Reproductive Technology (ART), gestational age will be determined by the date of embryo transfer or intrauterine insemination. For patients conceived naturally, gestational age will be determined from the menstrual history and confirmed by the fetal crown-rump length of the largest twin at the first-trimester ultrasound.

## 6.2 Cerclage group

Women allocated to a cervical cerclage will be receiving the intervention according to local protocol, within a week after randomisation. Briefly, 4 senior clinicians (Y.T.N.H., C.H.L., T.Q.B., N.T.V.), who had experienced with cerclage, will perform cervical cerclage, using Mc Donald technique, under spinal anaesthesia.

## 6.3 Pessary group

Pessary, a soft, flexible, silicone pessary, purchased from the manufacturer (Arabin, Dr Arabin GmbH & Co KG, Germany), will be inserted through the vagina, upward around the cervix by 4 senior clinicians (Y.T.N.H., C.H.L., T.Q.B., N.T.V.), who had experienced with pessary used, within one week of randomisation. The size of the pessary will be determined at the time of speculum inspection (Arabin and Alfirevic, 2013).

## 6.4 Cerclage plus progesterone group

In the cerclage plus progesterone group, 400 mg vaginal progesterone, purchased from the manufacturer (Cyclogest 400mg, Actavis, United Kingdom), will be applied once daily at bedtime, within two days after cerclage insertion. Participants will be asked to record their drug application in a patient diary sheet for up to 147 days.

## 6.5 Pessary plus progesterone group

In the pessary plus progesterone group, 400 mg vaginal progesterone, purchased from the manufacturer (Cyclogest 400mg, Actavis, United Kingdom), will be applied once daily at bedtime, within two days after pessary insertion, in addition to the pessary that has been placed. Participants will be asked to record their drug application in a patient diary sheet for up to 147 days.

## 6.6 Follow-up

In all groups, participants will be re-assessed at 14 days post-randomisation for any possible adverse event. After that, participants will be seen monthly or weekly per local protocol. CL measurement will not be performed routinely after randomisation, unless for patients’ preference. In case the CL was shortened, further intervention, if any, will be based on the clinician’s decision after a discussion with the patient. In case of premature rupture of the membranes, active vaginal bleeding, other signs of preterm labor or severe patient discomfort, the vaginal progesterone and pessary or cerclage, will be removed. If participants develop (threatened) preterm labor, they will receive treatment per local protocol. Intervention will be stopped at 37^0/7^ weeks of gestation or at delivery.

Compliance rate to progesterone will be calculated by dividing the number of progesterone doses used since the last visit by the number of progesterone doses that should have been used since the last visit. Women will be defined as compliant when the compliance rate are over 80%.

## 6.7 Quality control

Quality control of screening, handling of data, and verification of adherence to protocols at the different centers will be performed on a regular basis by the trial coordinators.

7. METHODS

## 7.1 Study endpoints

Primary endpoint

The primary endpoint will be PTB before 34 weeks of gestation for any indication

Secondary endpoints

Obstetrics outcomes including:

Fetal death before 24 weeks of gestation

Stillbirth will be diagnosed as a baby born with no signs of life at or after 28 weeks’ gestation (WHO).

Gestational age at delivery

Time from randomisation to delivery

PTB <24 weeks, <28 weeks, <32 weeks and <37 weeks

Spontaneous PTB <28 weeks, <34 weeks, <37 weeks

Iatrogenic PTB <28 weeks, <34 weeks, <37 weeks

Onset of labor: spontaneous, labor induction, elective C-section

Mode of delivery: vaginal delivery, C-section (elective, suspected fetal distress, non-progressive labor)

All livebirths at any gestational age

Use of tocolytic drugs

Use of antenatal corticosteroids

Use of MgSO4 for neuroprotection

Preterm prelabour rupture of membranes

Length of maternal admission for preterm labour (days)

Chorioamnionitis

Maternal side effects, including vaginal discharge, fever, vaginal bleeding, vaginal infection (confirmed by vaginal discharge culture), vaginal pain, pessary repositioning and necrosis or rupture of the cervix).

Maternal morbidity, including thromboembolic complications, urinary tract infection treated with antibiotics, pneumonia, endometritis, hypertensive disorder, eclampsia, haemo- lysis, elevated liver enzymes, low platelet count syndrome.

Maternal mortality

Neonatal outcomes, including:

Birthweight (mean), birthweight <1500 g, birthweight <2500 g

Congenital anomalies after randomisation

5-min Apgar score, 5-min Apgar score <7

Admission to neonatal intensive care unit (NICU)

Length of NICU admission (days)

Death before discharge

Perinatal death will be diagnosed as stillbirth or neonatal death of a baby of 20 or more completed weeks of gestation.

Respiratory distress syndrome (RDS), diagnosed as the presence of tachypnoea >60/minute, sternal recession and expiratory grunting, need for supplemental oxygen, and a radiological picture of diffuse reticulogranular shadowing with an air bronchogram (Hjalmarson, 1981).

Periventricular haemorrhage II B or worse, will be diagnosed by repeated neonatal cranial ultrasound by the neonatologist according to the guidelines on neuro-imaging described by de Vries et al.

Necrotizing enterocolitis (NEC) will be diagnosed according to Bell.

Proven sepsis, will be diagnosed on the combination of clinical signs and positive blood cultures.

Composite of poor perinatal outcomes, defined as foetal or neonatal death, intraventricular haemorrhage, respiratory distress syndrome, necrotizing enterocolitis or neonatal sepsis.

## 7.2 Sample size calculation

The PTB <34 weeks of gestation in women with a twin pregnancy and a cervix ≤28 mm in our previous AP study at My Duc hospital was 24.2% in the pessary group. In order to show or refute that cervical cerclage decreases the PTB rate by 50% (from 24.2% to 12.1%), we need to randomise 320 women (alpha level 0.05, power 80%). The statistical power to study the effect of progesterone by randomizing 320 women is 82% (alpha level 0.05, PTB rate in progesterone group 54.5%, absolute PTB rate difference= 15.5%). Considering a 5% lost to follow up and protocol violation we plan to recruit 340 participants (85 per arm).

## 7.3 Duration of the study

The study duration is estimated to be of 9 years, with final date of completion of patients’ recruitment in March 2027.

## 7.4 Statistical evaluation

Statistical analysis will be conducted according to the intention-to-treat principle, in which all randomised women will be considered in the primary comparison between treatment groups. The per-protocol analysis may be conducted, but these results would be considered exploratory only. All tests will be two-tailed, and differences with p-value <0.05 will be considered statistically significant.

In view of the two-by-two factorial design, the analysis will be done separately for cerclage versus pessary and for progesterone versus no progesterone. We will develop a separated detailed statistical analysis plan that will be completed prior to data-lock.

Baseline data

Baseline characteristics will be described by descriptive analysis, and the balance between the four arms will be assessed. For continuous variables, the normality test will be estimated using frequency histograms and the Shapiro test initially. If the parameters are normally distributed, they will be presented as mean with standard deviation (SD) and compared using student t-test or ANOVA test. If the parameters are non-normally distributed, their medians and inter-quantile ranges (IRQs) will be reported, and non-parametric tests will be utilized to test the distribution of these variables. For categorical variables, we will present the proportions of the two arms, and they will be compared using Pearson’s chi-square test or Fisher’s exact test where appropriate. In addition, we will also report the numbers of recruitment, participants lost to follow-up, protocols violation, and other relevant descriptive data.

Primary study parameter

The primary outcome, PTB before 34 weeks of gestation, will be compared using Pearson’s chi-square test or Fisher’s exact test for unadjusted analysis. We will also compute unadjusted risk ratio (RR) and its 95% confidence interval (95% CI). In the event of prominent imbalance of potential confounders between arms, we will perform multivariable Log-Binomial or Poisson Regression with robust variance estimate to compute adjusted RR and its 95% CI.

Secondary study parameter(s)

For continuous variables, results will be given as mean (standard deviation [SD]) and between-group differences will be assessed using Student’s t-test. For dichotomous endpoints, relative risk (RR) and 95% confidence interval (CI) values will be calculated. Time to delivery will be assessed using a Cox proportional hazard analysis and Kaplan-Meier estimates, where gestational age will be the time scale, birth will be the event and results will be compared with a log-rank test. Hazard ratio (HR) values will be estimated using a Cox proportional hazards model, with a formal test of the proportional hazards assumption. For neonatal outcomes, we will use cluster analysis taking into account the dependency between the twins.

Missing data and sensitivity analysis

For missing values regarding baseline characteristics, we will first perform analysis by excluding missing values; we will then perform multiple imputations to impute missing values and conduct subsequent analysis to estimate the robustness of the findings. For the loss of follow-up and protocol violation, we will attempt sensitive analyses to explore the effect of these factors on the trial findings.

Subgroup analysis

We plan a pre-specified subgroup analysis in women with a CL <25th percentile, and at the 25–50th percentile, 50–75th percentile and >75th percentile. The percentile will be determined based on the CL from all women after randomisation. We will test for interaction between CL and treatment effect on PTB <34 weeks and the composite of poor perinatal outcomes.

## 7.5 Data and safety monitoring board

We will establish an independent Data and Safety Monitoring Board (DSMB) to review and interpret data generated from the study and to review revisions of the protocol prior to their implementation. Its primary objectives are to ensure the safety of study subjects and the integrity of research data. The DSMB advises on research design issues, data quality and analysis, and research participant protections for the study.

| Prof. Joris van der Post | Chair of DSMB |
| --- | --- |
| Prof. Michael John Stark | Member of DSMB |
| Dr. Ewoud Schuit | Member of DSMB |

The DSMB will hold regular conference calls to review the protocol with respect to ethical and safety standards, monitor the safety of the trials, monitor the integrity of the data with respect to original study design, and provide advice on study conduct. The DSMB will review the progress of the trial, adjudicate adverse events, and decide on any premature closure of the study. The DSMB consists of three members. Voting members consist of individuals who are impartial, independent of the investigator(s), and who have no financial, scientific, or other conflict of interests with the study.

## 7.6 Interim anlysis

According to the first interim analysis in October 2021, which included complete data of 156 participants, the DSMC recommended continuing recruitment till complete assemblage of two thirds of the total sample’s data to undergo a second interim analysis.

8. SAFETY REPORTING

**WMO event**

The investigator will inform the subjects and the reviewing accredited medical research ethics committee; if anything occurs, on the basis of which it appears that the disadvantages of participation may be significantly greater than was foreseen in the research proposal. The study will be suspended pending further review by the accredited medical research ethics committee, except insofar as suspension would jeopardize the subjects’ health. The investigator will take care that all subjects are kept informed.

**Adverse and serious adverse events**

All observed or volunteered adverse events, regardless of treatment group or suspected causal relationship to intervention, will be recorded. Adverse events are defined as any undesirable experience occurring to a subject during a clinical trial, whether or not considered related to the intervention. All adverse events reported spontaneously by the subject or observed by the investigator, or his staffs will be recorded. A serious adverse event is any untoward medical occurrence or effect that at any dose results in death; is life threatening (at the time of the event); requires hospitalisation or prolongation of existing inpatients’ hospitalisation; results in persistent or significant disability or incapacity; is a congenital anomaly or birth defect; is a new event of the trial likely to affect the safety of the subjects, such as an unexpected outcome of an adverse reaction.

All SAEs will be reported to the accredited METC that approved the protocol, according to the requirements of that METC.

**Follow-up of adverse events**

All adverse events will be followed until they have abated, or until a stable situation has been reached. Depending on the event, follow up may require additional tests or medical procedures as indicated, and/or referral to the general physician or a medical specialist.

9. ETHICAL CONSIDERATIONS

**Regulation statement**

The study will be conducted according to the principles of the Declaration of Helsinki WORLD MEDICAL ASSOCIATION DECLARATION OF HELSINKI Ethical Principles for Medical Research Involving Human Subjects Version Edinburgh, Scotland, October 2000, with Note of Clarification on Paragraph 29 added by the WMA General Assembly, Washington 2002 and Note of Clarification on Paragraph 30 added by the WMA General Assembly, Tokyo 2004 and in accordance with the Medical Research Involving Human Subjects Act (WMO) and other guidelines, regulations and Acts.

The protocol will be sent for approval from Ethics Board of each participating hospital.

**Recruitment and consent**

The investigator must explain to each subject the nature of this study, its purpose, procedures, expected duration and the potential risks and benefits involved in study participation along with any discomfort it may entail. Each subject must be informed that participation in the study is voluntary, and that withdrawal of consent will not affect her right to the most appropriate medical treatment or affect the doctor relationship.

This informed consent will be given by means of a standard written statement. Informed consent will be written so as to be easily understood by the subject. The subject will be given the time to read and understand the statement herself before signing her consent and dating the document. The subject will receive a copy of the written statement once signed.

**Privacy aspects**

Participating subjects will be registered by a 5-digit number. This personal code will be on all forms retrieved from participants.

**Benefits and risks assessment, group relatedness**

There is insufficient evidence for a rational policy between the 2 strategies, arabin pessaries or cervical cerclage. Being cost-saving and simply placed could be potential benefits of arabin pessaries compared with cervical cerclage.

10. FEASIBILITY OF STUDY

Due to the low number of women with a twin pregnancy attending to the two hospitals from 2021, we extended the expected final date of the study in March 2027. In order to ensure adequate participant enrolment, all patients’ files will be pre-screened on the day of visit from 16^0/7^ to 22^0/7^ weeks of gestation in order to confirm eligibility.

11. ADMINISTRATIVE ASPECTS AND PUBLICATION

**Handling and storage of data and documents**

Data will be collected using a questionnaire and a software of SPSS.

Data monitoring will be done by principal investigator in My Duc hospital.

Data handling will be done anonymously, with the patient code only available to the local investigator. Patients will be asked for informed consent.

**End of study report**

The principal investigator will notify the accredited METC and the competent authority of the end of the study within a period of 90 days. The end of the study is defined as the last patient’s last visit. In case the study is ended prematurely, the principal investigator will notify the accredited METC within 15 days, including the reasons for the premature termination.

**Public disclosure and publication policy**

No specific arrangements will be made between any sponsors and the investigator concerning the public disclosure and publication of the research data. The principle investigator will publish the results of the study as soon as appropriate*.*

12. REFERENCES

1. Abdel-Aleem H, Shaaban OM, Abdel-Aleem MA. Cervical pessary for preventing preterm birth. Cochrane Database of Systematic Reviews 2013, Issue 5. Art. No.: CD007873. DOI: 10.1002/14651858.CD007873.pub3.
2. Adams TM. Does cervical cerclage decrease preterm birth in twin pregnancies with a short cervix. J Matern Fetal Neonatal Med. 2017 Apr 10:1-7.
3. Bell MJ. Neonatal necrotizing enterocolitis. Ann Surg 1978, 187:1–7.
4. de Vries LS, Eken P, Dubowitz LM: The spectrum of leukomalacia using cranial ultrasound. Behav Brain Res 1992, 49:1–6.
5. Dang QV, Nguyen KL, Pham DT, He TNY, Vu NK, Phan TNM, Le QT, Vuong TNL, Mol B. Cervical pessary versus vaginal progesterone for the prevention of preterm birth in women with a twin pregnancy and a cervix <38 mm: a randomized controlled trial. 2018;218(Supp1):S603-S4
6. Giedion A, Haefliger H, Dangel P. Acute pulmonary X-ray changes in hyaline membrane disease treated with artificial ventilation and positive end-expiratory pressure (PEP). *Pediatr Radiol* 1973; **1:** 145–52.
7. Goya M, Rodo C, Muñoz B, Juan M, Calle M, Serrano A, Pratcorona L, Carreras E. Cervical pessary to prevent preterm birth in twin’s pregnant women with a short cervix: a multicentre randomised controlled trial (PECEP-Twins). 13th World Congress of Fetal Medicine 2014.
8. [Poon LC](http://www.ncbi.nlm.nih.gov/pubmed/?term=Poon%20LC%5BAuthor%5D&cauthor=true&cauthor_uid=26990136), [Ciarlo M](http://www.ncbi.nlm.nih.gov/pubmed/?term=Ciarlo%20M%5BAuthor%5D&cauthor=true&cauthor_uid=26990136), [Kim E](http://www.ncbi.nlm.nih.gov/pubmed/?term=Kim%20E%5BAuthor%5D&cauthor=true&cauthor_uid=26990136), [Guzman ER](http://www.ncbi.nlm.nih.gov/pubmed/?term=Guzman%20ER%5BAuthor%5D&cauthor=true&cauthor_uid=26990136), [Nicolaides KH](http://www.ncbi.nlm.nih.gov/pubmed/?term=Nicolaides%20KH%5BAuthor%5D&cauthor=true&cauthor_uid=26990136). Cervical cerclage for preterm birth prevention in twin gestations with short cervix: a retrospective cohort study. Ultrasound Obstet Gynecol. 2016 Mar 16. doi: 10.1002/uog.15918. [Epub ahead of print].
9. Jarde A. Preterm birth prevention in twin pregnancies with progesterone, pessary, or cerclage: a systematic review and meta-analysis. BJOG. 2017 Jul;124 (8):1163-1173.
10. Jobe AH, Bancalari E: Bronchopulmonary dysplasia. Am J Respir Crit Care Med 2001, 163:1723–1729.
11. Liem S, Schuit E, Hegeman M, Bais J, Boer K, Bloemenkamp K, Brons J, Duvekot H, Bijvank B, Franssen M, Gaugler I, de Graaf I, Oudijk M, Papatsonis D, Pernet P, Porath M, Scheepers L, Sikkema M, Sporken J, Visser H, van Wijngaarden W, Woiski M, van Pampus M, Mol BW and Bekedam D. Cervical pessaries for prevention of preterm birth in women with a multiple pregnancy (ProTWIN): a multicentre, open-label randomised controlled trial. The Lancet. Published Online August 5, 2013 http://dx.doi.org/10.1016/ S0140-6736(13)61408-7.
12. Ment LR, Bada HS, Barnes P, Grant PE, Hirtz D, Papile LA, Pinto-Martin J, Rivkin M, Slovis TL: Practice parameter: neuroimaging of the neonate: report of the Quality Standards Subcommittee of the American Academy of Neurology and the Practice Committee of the Child Neurology Society. Neurology 2002, 58(12):1726–1738.
13. Nicolaides KH, Syngelaki A, Poon LC, de Paco Matallana C, Plasencia W, Molina FS, Picciarelli G, Tul N, Celik E, Lau TK, Conturso R. [Cervical pessary placement for prevention of preterm birth in unselected twin pregnancies: a randomized controlled trial.](http://www.ncbi.nlm.nih.gov/pubmed/26321037) Am J Obstet Gynecol. 2016 Jan;214(1):3.e1-9. doi: 10.1016/j.ajog.2015.08.051. Epub 2015 Aug 28.
14. Rode L., Klein K., Nicolaides K.H., Krampl-Bettelheim E. and Tabor A for the PREDICT group. Prevention of preterm delivery in twin gestations (PREDICT): a multicenter, randomized, placebo-controlled trial on the effect of vaginal micronized progesterone. Ultrasound Obstet Gynecol 2011; 38: 272–280 Published online in Wiley Online Library (wileyonlinelibrary.com). DOI: 10.1002/uog.9093.
15. Romero R, Nicolaides K, Conde-Agudelo A, Tabor A, O'Brien JM, Cetingoz E, et al. Vaginal progesterone in women with an asymptomatic sonographic short cervix in the midtrimester decreases preterm delivery and neonatal morbidity: a systematic review and metaanalysis of individual patient data. American journal of obstetrics and gynecology. 2012;206(2):124.e1-19.
16. [Saccone G](http://www.ncbi.nlm.nih.gov/pubmed/?term=Saccone%20G%5BAuthor%5D&cauthor=true&cauthor_uid=25644964), [Rust O](http://www.ncbi.nlm.nih.gov/pubmed/?term=Rust%20O%5BAuthor%5D&cauthor=true&cauthor_uid=25644964), [Althuisius S](http://www.ncbi.nlm.nih.gov/pubmed/?term=Althuisius%20S%5BAuthor%5D&cauthor=true&cauthor_uid=25644964), [Roman A](http://www.ncbi.nlm.nih.gov/pubmed/?term=Roman%20A%5BAuthor%5D&cauthor=true&cauthor_uid=25644964), [Berghella V](http://www.ncbi.nlm.nih.gov/pubmed/?term=Berghella%20V%5BAuthor%5D&cauthor=true&cauthor_uid=25644964). Cerclage for short cervix in twin pregnancies: systematic review and meta-analysis of randomized trials using individual patient-level data. Acta Obstet Gynecol Scand. 2015 Apr;94(4):352-8. doi: 10.1111/aogs.12600. Epub 2015 Mar 1.
17. Saccone G. Cervical pessary for preventing preterm birth in twin pregnancies with short cervical length: a systematic review and meta-analysis. J Matern Fetal Neonatal Med. 2017 Dec;30(24):2918-2925.
18. Schuit E, Stock S, Rode L, Rouse DJ, Lim AC, Norman JE, et al. Effectiveness of progestogens to improve perinatal outcome in twin pregnancies: an individual participant data meta-analysis. BJOG : an international journal of obstetrics and gynaecology. 2015;122(1):27-37.
19. De Vries LS, Eken P, Dubowitz L. The spectrum of leukomalacia using cranical ultrasound. Behav Brain Res. 1992;49(1) 1-6
20. <http://www.who.int/maternal_child_adolescent/epidemiology/stillbirth/en/>
